# PhaseDancer: a novel targeted assembler of segmental duplications unravels the complexity of the human chromosome 2 fusion going from 48 to 46 chromosomes in hominin evolution

**DOI:** 10.1186/s13059-023-03022-8

**Published:** 2023-09-11

**Authors:** Barbara Poszewiecka, Krzysztof Gogolewski, Justyna A. Karolak, Paweł Stankiewicz, Anna Gambin

**Affiliations:** 1 Faculty of Mathematics, Informatics, and Mechanics, University of Warsaw, Banacha 2, 02-097 Warsaw, Poland; 2 Department of Molecular and Human Genetics, Baylor College of Medicine, 1 Baylor Plaza, 77030 Houston, TX USA; 3 Chair and Department of Genetics and Pharmaceutical Microbiology, Poznan University of Medical Sciences, 60-806 Poznan, Poland

**Keywords:** De-novo assembly, Segmental duplications, Long-read PacBio sequencing, Chromosomal fusion, Complex genomic rearrangements

## Abstract

Resolving complex genomic regions rich in segmental duplications (SDs) is challenging due to the high error rate of long-read sequencing. Here, we describe a targeted approach with a novel genome assembler PhaseDancer that extends SD-rich regions of interest iteratively. We validate its robustness and efficiency using a golden-standard set of human BAC clones and in silico-generated SDs with predefined evolutionary scenarios. PhaseDancer enables extension of the incomplete complex SD-rich subtelomeric regions of Great Ape chromosomes orthologous to the human chromosome 2 (HSA2) fusion site, informing a model of HSA2 formation and unravelling the evolution of human and Great Ape genomes.

**Supplementary Information:**

The online version contains supplementary material available at 10.1186/s13059-023-03022-8.

## Background

Continuous improvement of sequencing technologies along with the development of efficient computational assembly approaches have facilitated better understanding of genome evolution and architecture [1–3]. Segmental duplications (SDs) have been shown to be one of the key factors catalysing the dynamic evolutionary rearrangements of the genomes, particularly in primates [4–6]. Importantly, analyses of the most recent human genome reference build (except chromosome Y) [7] by the Telomere-to-Telomere (T2T) Consortium have revealed that 7% of the human genome consists of SDs (218 Mb of 3.1 Gb) [8].

**Fig. 1:**
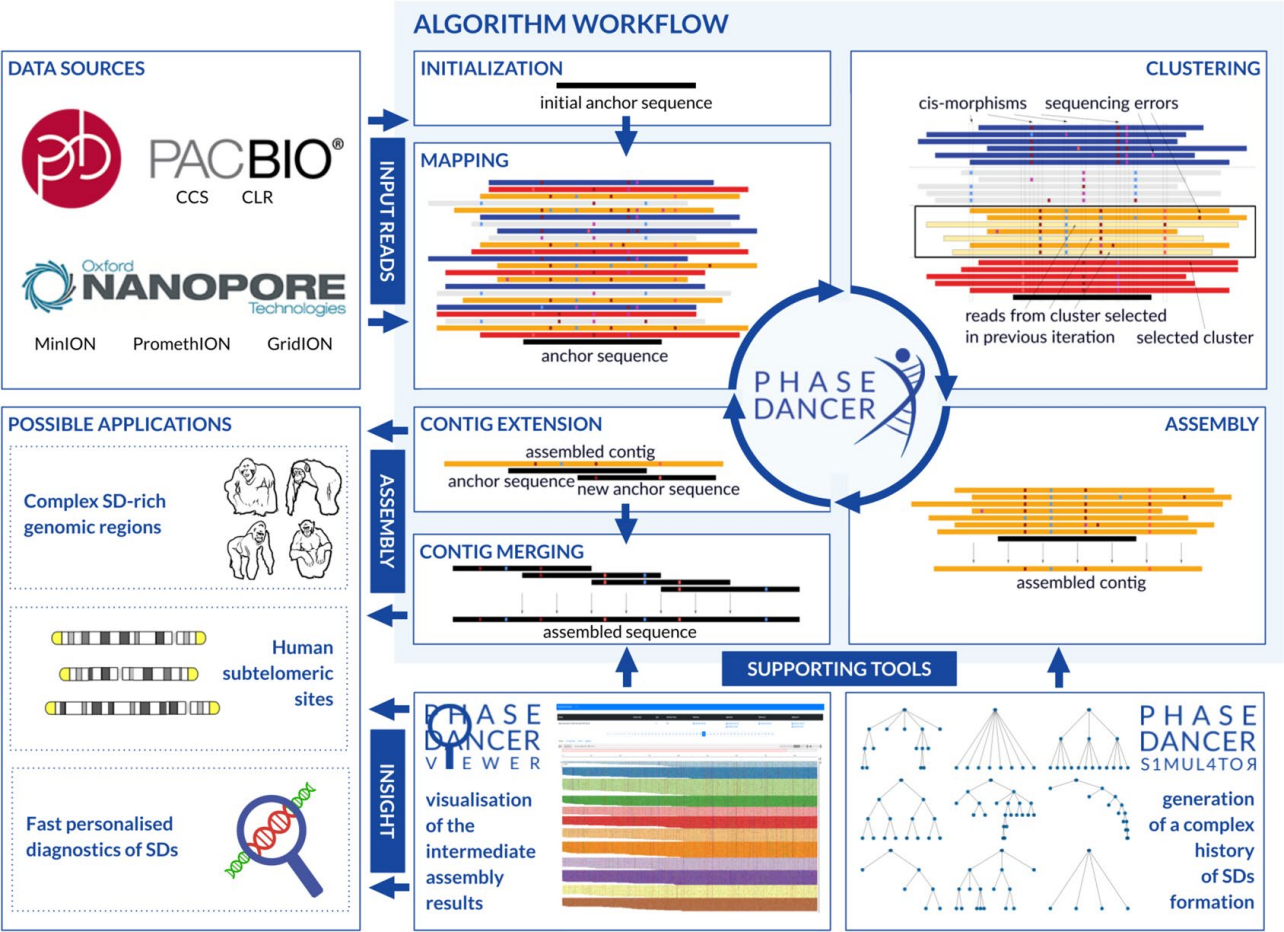
A workflow of the PhaseDancer algorithm and the accompanying tools. PhaseDancer works with next generation sequencing long-read data e.g. Oxford Nanopore or PacBio. Starting with an initial anchor sequence, the core workflow of PhaseDancer iterates along four major steps: (i) mapping the reads on the anchor sequence, (ii) clustering the mapped reads and selection of a cluster with the reads originating from the genomic region represented by the anchor sequence, (iii) assembling these reads into a contig, and (iv) extending the current anchor sequence using the contig to a new anchor sequence processed in the next iteration. After all iterations, the algorithm outputs the final assembled sequence. PhaseDancer is also accompanied with two supporting tools - the semi-supervised character of PhaseDancer is complemented by PhaseDancerViewer that enables the intermediate control of assembly process, whereas PhaseDancerSimulator generates in silico data for profound validation of the algorithm. Thanks to its high efficiency, PhaseDancer can be used for resolving challenging genomic tasks, involving segmental duplication (SD) assembly

Assembly of SD-rich genomic regions has been one of the most important computational challenges in building a reference haploid genome [8]. Thus far, a number of general purpose assemblers have been developed, e.g. FALCON [9], Miniasm [10], Canu [11], Flye [12], Wtdbg2 [13], Shasta [14], and HiCanu [15]. Additionally, SDA assembler has been specifically dedicated to resolve SDs [16]. Currently, the high error rate of next generation sequencing (NGS) long-read data leaves a significant fraction of the unassembled regions mainly corresponding to SDs and necessitating application of targeted methods (Fig. 1). To date, only assemblies from Ultra-Long Oxford Nanopore (UL ONT) or high-quality PacBio circular consensus sequencing (CCS) reads have been validated successfully on the data sets enriched with SDs; however, these technologies are still limited by their high cost [17]. Technologies generating reads of length shorter than UL ONT or lower accuracy than PacBio CCS (HiFi) have turned out insufficient to accomplish these tasks [18].

**Fig. 2:**
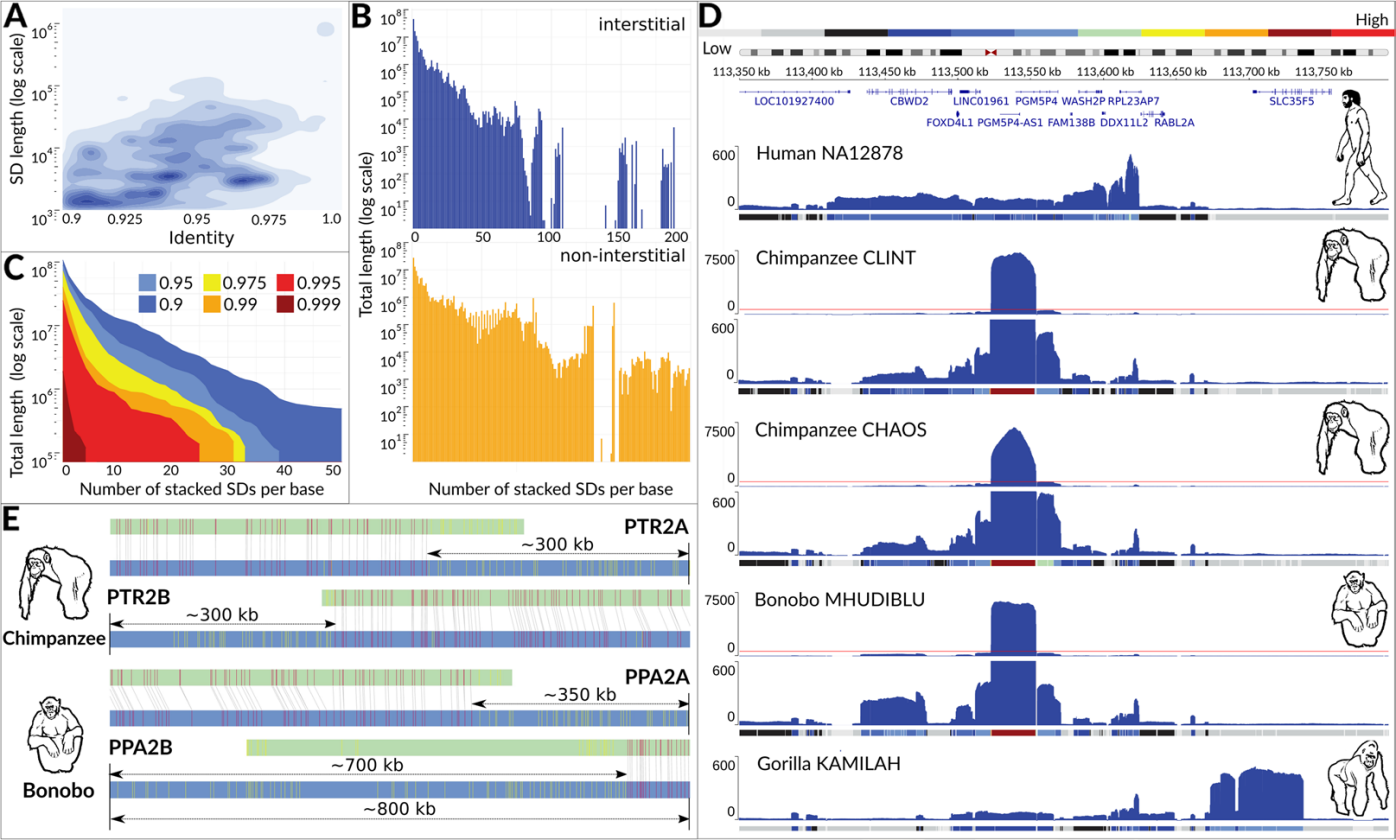
An overview of SDs characteristics and the study motivation. Based on the most recent T2T human genome assembly: **A** A contour plot of the SD abundance given their sequence identity (90–100%, x axis) and the total length (Mb, y-axis, log-scale), where the blue colour intensifies with the increasing number of SDs; **B** A barplot of the SDs total length (Mb, log-scale, y-axis) given the total number of SDs copies (x-axis) located at the interstitial (top, blue) and non-interstitial (bottom, yellow) genomic regions; **C** An area plot of the SDs’ total length (Mb, log-scale, y-axis) for SDs with at least given number of copies (x-axis) and the minimal percent of sequence identity (area colour). Here, the number of stacked SDs per base is the number of reads overlapping a given base position of the reference genome. **D** A normalised depth-of-coverage histogram of the aligned whole-genome circular consensus sequencing (CCS) reads in the human (NA12878), two chimpanzees (Clint, Chaos), bonobo (Mhudilbu), and gorilla (Kamilah) genomic regions syntenic to those flanking the HSA2 fusion site. For bonobo and both chimpanzees two depth-of-coverage tracks are shown. The top track presents the full scale of all data, whereas the bottom track zooms-in the coverage of values excluding the extremely high coverage region. The red line on each of the top tracks indicates the y-axis limit of the bottom track. Note the high coverage of the ~31 kb fragment previously found to be amplified about 400 times in the chimp genome [19]. **E** Optical genome mapping was used to assess the current incompleteness of the subtelomeric assemblies in chimpanzee and bonobo genomes (panTro5, panTro6, and panPan3). Each of the subtelomeric ends was estimated to lack at least 0.3 Mb of the DNA sequence

Importantly, given that the most recent T2T human genome assembly contains approximately 7% SDs and that SD-rich human chromosome 2 (HSA2) syntenic sites in Great Apes reference genomes are incomplete, a more efficient approach to resolve their structure is needed (Fig. 2).

We developed PhaseDancer, a novel, fast, and robust assembler that follows a locally-targeted approach to resolve SD-rich complex genomic regions. The tool is designed to work with long-reads (ONT, PacBio) and tuned for error-prone data (Fig. 1). Based on the iterative approach with randomised clustering procedure, the workflow of PhaseDancer enables the extension of a user-provided initial sequence contig even from complex genomic regions. To assess its performance, we validated PhaseDancer using bacterial artificial chromosome (BAC) clones sampled from the known SDs as well as computationally simulated sequences reflecting a complex evolutionary history of SDs. To demonstrate the efficacy and biological utility of PhaseDancer, we assembled subtelomeric regions of chromosomes 2Apter, 2Bpter, 9pter, 12pter, and 22qter in bonobo, chimp, gorilla, and orangutan together with a syntenic complex SD-rich site of HSA2 fusion that reduced the number of chromosomes from 48 in Great Apes to 46 in *Homo sapiens*, Neandertals, and Denisovans [20–23]. Based on our assembled sequences, we have proposed a novel evolutionary model for complex HSA2 formation, indicating the most plausible key mutational events.

## Results

### Design and implementation of PhaseDancer

In contrast to the existing long-read assemblers that follow the *top-down* paradigm and operating simultaneously on all available reads, we implemented an approach with contigs generated in a bottom-up manner, working with a gradually expanded set of sufficiently similar reads. As a result, our de novo assembler can generate several Mb long contigs enriched with SDs.

The algorithm implements an iterative strategy for extending the *initial anchor* sequence by finding the best fitting set of reads to expand the processed *anchor* sequence.

Due to the efficient integration of the state-of-the-art components used in the workflow (Methods), PhaseDancer generates contigs with the fragments repeated up to several dozens times in the genome with at least 0.1% divergence. The preprocessing time of 200 GB FASTQ data is approximately one hour. The conducted runtime experiments have proven that PhaseDancer is a fast and robust assembler. For example, the targeted assembly of a 1 Mb SD contig (coverage 40x, sequencing error 15%, average read length 18 kb with a standard deviation 3 kb) took on average 20 minutes on the server with 56 Intel(R) Xeon(R) E5-2690 v4 @ 2.60GHz CPUs (Methods).

PhaseDancer is accompanied by two supporting tools, PhaseDancerViewer and PhaseDancerSimulator. PhaseDancerViewer visualises the intermediate results of each algorithm iteration and enables running the assembler in a monitored and semi-supervised fashion, facilitating the PhaseDancer parameters tuning. PhaseDancerSimulator generates in silico SD sequences, resulting from various scenarios of a parameter-controlled evolutionary processes. Such synthetic data provide a broad scope of model testing and verification strategies with the a priori known dataset.

### Validation on SD-rich human BAC clone sequences

To validate the PhaseDancer assembly quality, first, we used a set of BAC clones from haploid CHM13hTERT human cell line (sequenced using PacBio RS II; coverage 45x, N50 20,000), considered as a gold-standard for a validation and benchmarking [7]. We employed a validation pipeline commonly used to measure the quality of assemblies on such data; available at https://github.com/skoren/bacValidation [14, 15]. This pipeline evaluates two measures describing the quality of the assembly of the BAC clones sequences: (i) resolving success (BAC clone is considered as resolved if an alignment covers 99.5% of its length), (ii) alignment accuracy (measured as a median of the Phred Quality Scores (*Q*) [24] of the alignment identity of the resolved BAC clones). The score *Q* quantifies the probability (*p*) of an incorrect base call as $p = 10^{-\frac{Q}{10}}$.

PhaseDancer performance was compared with the results obtained from the Flye and Wtdgb2 assemblers that work with the error-prone PacBio reads. Out of 341 BAC clones studied, PhaseDancer resolved 292 clones (85.5%, median Phred Quality Value: 26.81), whereas Flye and Wtdgb2 resolved 91 (26.69%, med. 36.48) and 77 clones (22.58%, med. 30.07), respectively. Importantly, after backtracking of the PhaseDancer failures, we established that the unresolved BAC clones represented either SD regions with low-coverage or SDs enriched in tandem repeats.

### In silico verification and benchmarking

To evaluate the accuracy of the PhaseDancer performance, we tested the quality of the assembled sequences from the collapsed SDs generated by PhaseDancerSimulator. We simulated the collapsed SDs using 10 different evolutionary scenarios: flat with two, four, and eight leaves; three types of bifurcating; cascading with four and eight leaves; and two random with 10 leaves (Table 1). PhaseDancerSimulator was run with the above-mentioned set of parameters. Additionally, for each of the simulated SDs, random sequences were added at their beginnings and ends. Unique random sequences preceding each collapsed SD portion of the generated sequences were used as an initial anchor sequence for the assembly process.

**Table 1:** Assessment of the SDs assembly quality for different tools (columns) in various evolutionary topologies generated by PhaseDancerSimulator (rows). For each tool and toplogy: (i) upper cell contains Phred Quality Score (*Q*) - the larger value the lower error frequency in the assembled sequences; (ii) lower cell contains a percent of correctly resolved SDs (the expected are sequences from the leaves of the assessed topology). The comparison was evaluated for the following parameters setting of PhaseDancerSimulator: coverage 40x, sequencing error 15%, SD sequence identity 99.5%, average read length 18 kb, read length standard deviation 3 kb, and the simulated SD contig size 0.5 Mb. Timeout - the computation time exceeded 96 hours; N/A - not available, the assembly process failed; ^a^ four shorter SDs were assembled instead of the two excepted

SDs History	PhaseDancer	Canu	Miniasm	Flye	Wtdbg2	SDA
	29.42	20.73	9.19	23.05	17.80	30.21
	100.0%	50.0%	50.0%	50.0%	50.0%	0%^a^
	30.19	21.64	8.92	22.91	17.65	30.41
	100.0%	25.0%	25.0%	25.0%	25.0%	100%
	30.26	20.76	8.92	22.89	-	-
	100.0%	12.5%	12.5%	12.5%	0.0%	Timeout
	30.14	18.74	8.85	20.27	-	-
	100.0%	12.5%	12.5%	12.5%	0.0%	Timeout
	30.08	18.71	8.76	-	-	-
	100.0%	8.3%	8.3%	N/A	0.0%	N/A
	29.83	17.34	8.53	17.901	-	-
	100.0%	12.5%	12.5%	12.5%	0.0%	Timeout
	30.10	19.63	8.91	21.25	17.19	-
	100.0%	25.0%	25.0%	25.0%	25.0%	Timeout
	30.24	18.25	8.45	18.22	-	-
	100.0%	12.5%	12.5%	12.5%	0.0%	Timeout
	30.13	18.90	8.59	-	-	-
	100.0%	8.3%	8.3%	N/A	0.0%	N/A
	30.04	17.93	8.55	-	-	-
	100.0%	8.3%	8.3%	N/A	0.0%	N/A

^a^SDA assembled four contigs instead of the expected two. However, two pairs of them form one allelic variant

On such generated synthetic datasets, PhaseDancer was benchmarked against the several commonly used assemblers supporting error-prone NGS long reads: Canu [11], Wtdbg2 [13], Flye [12], Miniasm [10], and SDA [16] (Table 1).

To compare the assembly quality of the above tools, we calculated the Levenshtein distance between all assembled contigs and the simulated SDs. Next, for each assembled contig, we assigned the simulated SD for which: (i) the alignment covers at least 95% of the contig length, and (ii) the alignment Phred Quality Score was highest among all SDs. This assignment procedure allowed us to determine the number of the resolved simulated SDs generated by each assembler.

**Fig. 3:**
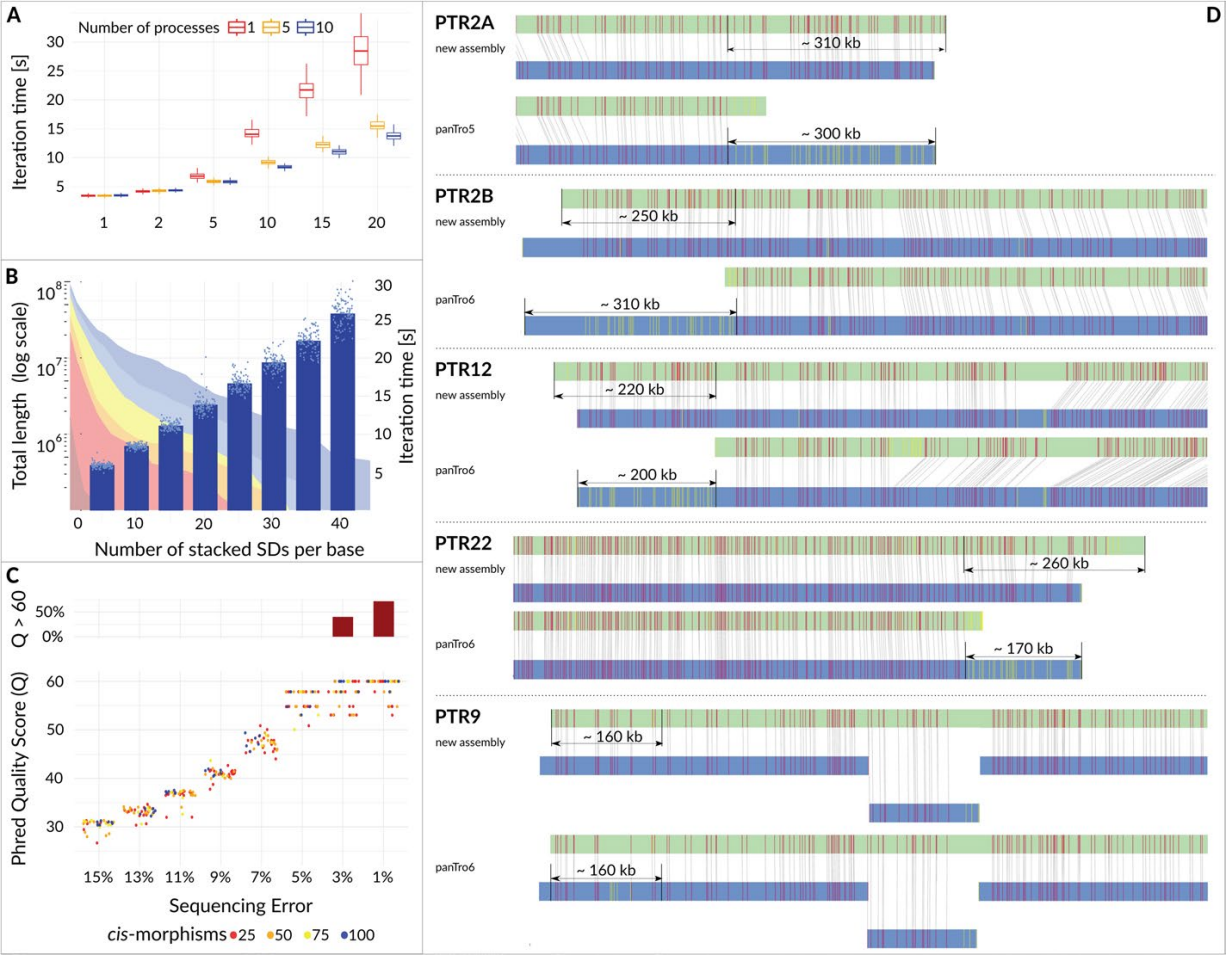
Time complexity, feasibility, and correctness of PhaseDancer. **A** Computational time performance (y-axis) for different number of stacked SDs (x-axis) and processes (colour scale). Each boxplot represents 100 iterations of PhaseDancer for a given setting. **B** Feasibility space for SDs in human. PhaseDancer resolves all SDs with the number of stacked SDs per base as for SDs identified by T2T human genome assembly (area plot, Fig. 2C). For a given number of stacked SDs (x-axis) the height of each bar indicates an average runtime of PhaseDancer iteration (right y-axis) along with a standard deviation (error bars) and individual measurements (points). **C** The evaluation of PhaseDancer assemblies using the Phred Quality Score (Q; y-axis). The samples used for evaluation were generated by PhaseDancerSimulator, with fixed parameters including a coverage of 40x, an average read length of 18 kb, and a read length standard deviation of 3 kb. The x-axis represents different sequencing error levels, while the colour scale indicates different numbers of cis-morphisms per 10 kb window. The additional upper panel in the figure shows the percentage of assembly tasks with no errors (Q > 60) using bar plots. Remarkably, our analyses revealed no significant changes in assembly quality for different PhaseDancerSimulator topologies (SDs evolutionary scenarios). **D** Correctness of the PhaseDancer assemblies was assessed using optical genome mapping (OGM). All HSA2 syntenic sites of the chimpanzee genome were in concordance with the corresponding OGM molecules (BssSI enzyme shown)

PhaseDancer has successfully resolved all of the simulated SDs with no alignment of Phred Quality Score lower than 29 (accuracy over 99.874%). Other assemblers managed to resolve at most one reference SD per scenario (with one exception, where SDA resolved two SD copies scenario), and only Canu [11], and Miniasm [10] produced one sequence for all scenarios. Flye [12] resolved one simulated SD only for models consisting of up to eight SDs, whereas Wtdbg2 [13] resolved only up to four SDs. Some assemblers failed to complete their assembly task either due to exceeding the 96-hour time limit or execution error during the assembly process (Table 1). Additionally, we broadly assessed the PhaseDancer performance on in silico reads of various properties provided by PhaseDancerSimulator (Fig. 3).

### Unveiling HSA2 fusion event

Following the successful validation of PhaseDancer, we applied our algorithm to the unresolved subtelomeric regions of the selected chromosomes in Great Apes i.e chimpanzee, bonobo, gorilla, and orangutan, syntenic to HSA2, to unravel the mechanism of reduction of the chromosome number during human speciation after divergence from chimpanzee/bonobo. These regions likely reflect high similarity with the ancestral chromosomes 2Apter and 2Bpter, that might have predisposed them for the evolutionary chromosomal fusion event.

Based on classical cytogenetics [22, 25–27] and molecular methods [28–35], HSA2 was proposed to have arisen as a product of the end-to-end fusion of telomeric repetitive sequences of the ancestral primate chromosomes 2Apter and 2Bpter. Subsequently, the unstable dicentric chromosome was rescued by a loss of satellite DNA sequences in the vestigial centromere at 2q21.2 [26–28, 30, 36–40]. Prior to the fusion, both ancestral chromosomes 2A and 2B underwent ancestral large pericentric inversions, before the chimp-gorilla lineage split [25, 29, 31, 40] and after the orangutan-gorilla divergence [25, 29, 31, 41], respectively (Additional file 1: Fig. S1).

**Fig. 4:**
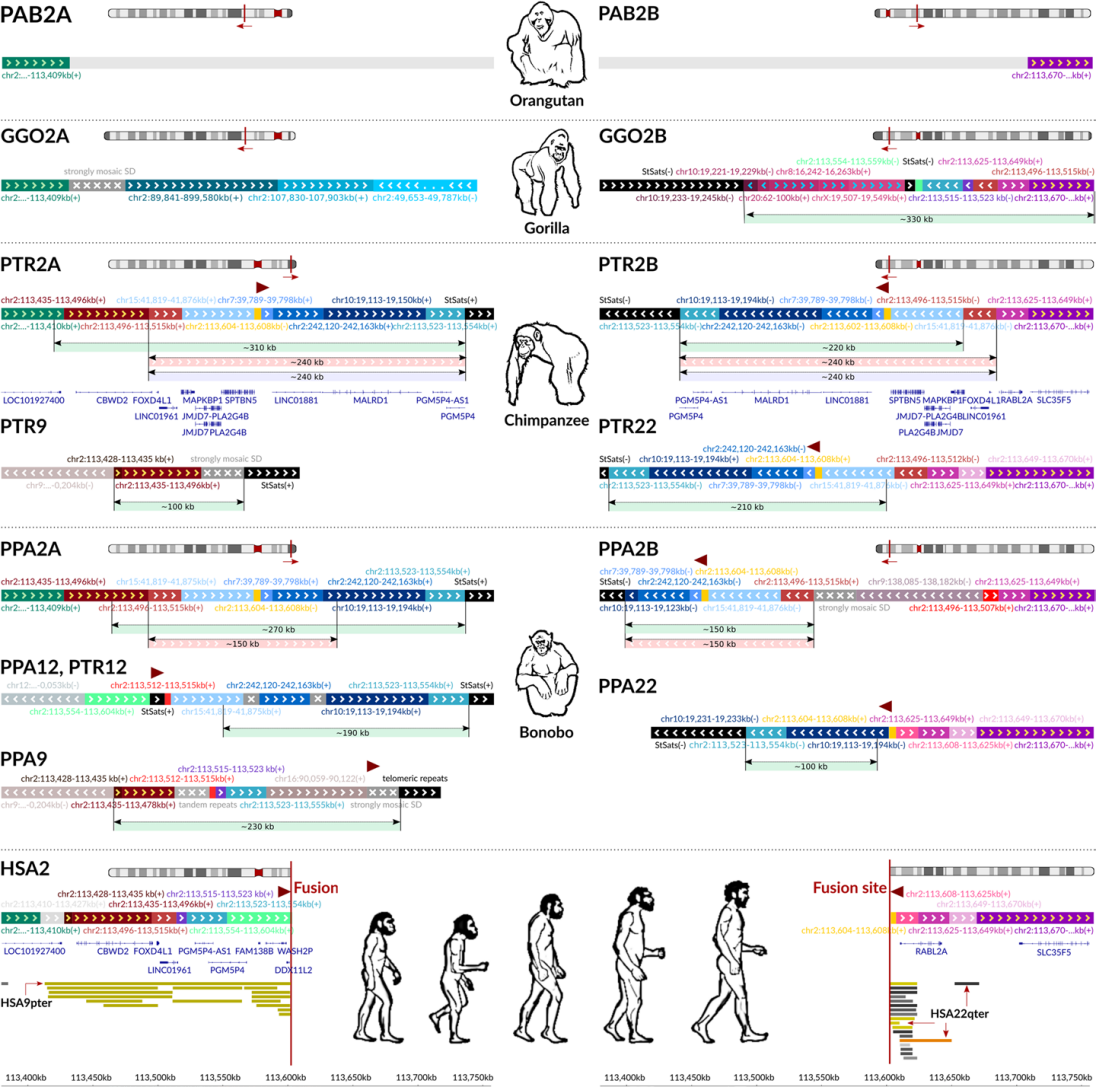
Genome architecture flanking the HSA2 fusion site and the syntenic genomic regions in Great Apes and human. From the top, the figure depicts the sequences from: orangutan (PAB) and gorilla (GGO) chromosomes 2Apter and 2Bpter; chimpanzee (PTR) and bonobo (PPA) chromosomes 2Apter, 2Bpter, 9pter, 12pter and 22qter; and human HSA2, all together with the corresponding coding regions track. Each individual contig is represented by a uniquely coloured stripe consistent among species/chromosomes, labelled with the coordinates with respect to the human genome build (hg38) and designated with the arrowheads indicating the DNA strand. Dark grey contigs with white crosses depict strongly mosaic SDs or tandem repeats that cannot be graphically presented in a legible way. Brown arrowheads depict the TAR1 satellite and degenerate telomeric repeats at the HSA2 fusion site and their orthologs in Great Apes. Below each contig assembly a coloured stripe depicts: (i) green - the novel reconstructed assembly along with an approximate size, (ii) pink - the high homology region between chromosomes 2Apter and 2Bpter presumably triggering the fusion event, and (iii) light blue - the region that was lost after the fusion event with respect to the HSA2. HSA2 is also equipped with a track of collapsed SDs including ~190 kb fragment homologous to HSA9pter and three fragments ~68 kb in size in total homologous to HSA22pter. The azure contig (chr2:113,523-113,554 kb) was found to be amplified ~400 times in the chimpanzee genome [19]

We confirmed the incompleteness and partial incorrectness of the latest genome builds of the subtelomeric sequences of Great Apes chromosomes 2Apter and 2Bpter using optical genome mapping (OGM) (Additional file 1: Figs. S2-S5) and direct sequence analysis (Additional file 1: Figs. S6, S7). The uniqueness and non-recurrence of this event was validated by analysing the human population SNV and SV polymorphisms flanking the HSA2 fusion site (Fig. 4, Additional file 1: Tables S1, S2 and Fig. S8).

### Great Apes genomes analysis

**Orangutan.** The orangutan genome differs from gorilla genome by one and from chimpanzee, bonobo, and human genomes by two gross chromosomal inversions rearranging them from acrocentric to submetacentric chromosomes. Using PhaseDancer in combination with OGM, we confirmed that the regions syntenic to the HSA2 fusion site map to the latest orangutan genome build showing any structural variations (Additional file 1: Fig. S2).

**Gorilla.** PhaseDancer generated assembly extending GGO2Bpter with ~330 kb, reaching the highly repetitive subtelomeric satellites (StSats) regions. The novel fragment of the GGO2Bpter is homologous to the proximal side of the HSA2 fusion. However, inside this fragment, we identified an ~54 kb sequence homologous to the distal side of the HSA2 fusion (chr2:113,496-113,550 kb). The ~44 kb contig on GGO2Apter (Fig. 4, the grey contig) is a region that maps to many different locations not related to the fusion site. Using OGM, we confirmed the presence of an erroneously scaffolded ~89 Mb region in the latest GorGor6 assembly (Additional file 1: Fig. S3).

**Chimpanzee.** Using OGM, we found false positive breakpoints on PTR2Apter in the latest chimpanzee chromosome build (panTro6) that resulted in placing the subtelomeric region interstitially, whereas no errors were found in the PTR2Bpter subtelomeric region (Additional file 1: Fig. S4). PhaseDancer extended both PTR2Apter and PTR2Bpter with an ~270 kb sequence, reaching StSats repetitive sequences, each harbouring ~240 kb of the fully homologous fragments. Importantly, the detected homologies encompass a fragment of ~190 kb that was likely deleted during the fusion event, whereas the remaining ~68 kb fragment is homologous to HSA2 near the fusion site. By homology to the human reference genome (including chromosomes 2, 7, 10, and 15), on the deleted fragments we annotated six coding genes: *MAPKBP1*, *JMJD7*, *PLA2G4B*, *JMJD7-PLA2G4B*, *SPTBN5*, and *MALRD1* and three lncRNAs *LINC01881,* *LINC01961*, and *PGM5P4-AS1*. All coding regions were subjected to the downstream transcriptomic analyses and their activity was assessed in different locations of human brain using the RNA-seq transcriptomic data [42] (Methods and Additional file 1: Fig. S9).

Interestingly, we found a strong homology between the region chr2:113,554-113,604 kb next to the fusion site and the chimpanzee subtelomeric region at PTR12pter and extended this region towards StSats. As a result, we identified an ~168 kb homology of PTR12pter to both PTR2Apter and PTR2Bpter, adjacent to an ~31 kb fragment that was found to be amplified ~400 times in the chimpanzee genome [19] and homologous also to the region near the HSA2 fusion site (chr2:113,523-113,554 kb; Figs. 2D, 4). Similarly, sequence homology between the human chromosomal region chr2:113,625-113,670 kb and the chimpanzee subtelomeric region at chromosome 22q led us to explore PTR22qter. The assembled fragment encompasses greater than 240 kb highly homologous fragment between PTR22qter and PTR2Apter and PTR2Bpter, adjacent also to the above-mentioned ~31 kb fragment (as in PTR12pter).

**Bonobo.** Analogously to the above Great Apes, both PPA2Apter and PPA2Bpter subtelomeric regions were validated using OGM (Additional file 1: Fig. S5) and were extended to the StSats repetitive sequences by ~270 kb and ~120 kb, respectively. Approximately 150 kb of homology was detected between these chromosomes and the fragments of ~190 kb and ~280 kb from PPA2Apter and PPA2Bpter, respectively, were found to be absent on HSA2. Similarly to the chimpanzee genome, because of the discovered homology between HSA2 fusion site and the bonobo chromosomal regions PPA9pter, PPA12pter, and PPA22qter, we assembled their subtelomeric regions revealing strong homologies to PPA2Apter and PPA2Bpter. However, an extension of PPA9pter from the ~61 kb homology region with HSA2 towards StSats confirmed an additional homology (separated by an insertion) to the above-mentioned ~31 kb fragment amplified in chimpanzee (Figs. 2D, 4) [19]. Similarly to chimpanzee genome, the selected transcripts were analysed to determine genes distinguishing the species (Additional file 1: Fig. S9, Methods). Using OGM, we independently validated the presented novel assemblies, extending the current reference genomes of bonobo, chimpanzee, and gorilla, generated using PhaseDancer (data shown for chimpanzee, Fig. 3D).

**Human.** Finally, using PhaseDancer, we assembled the NGS data from ten individuals from the Human Pangenome Project, T2T Diversity Panel [43] and three individuals from the Genome in the Bottle project [44]. The selected individuals represent five main human superpopulations: African, admixed American, East Asian, European, and South Asian. In particular, we assessed the polymorphisms of the 5 kb region directly flanking the HSA2 fusion site. The selected sequences corresponding to the region were subjected to the downstream analyses using RepeatMasker and multialigned to detect any possible genomic variety. No significant structural variations (i.e. duplications, deletions, inversions, indels) were detected (Additional file 1: Tables S1, S2 and Fig. S8).

### Analyses of the newly assembled two chimpanzee genomes

To confirm the structure of the assembled genomic extensions obtained using PhaseDancer, we incorporated additional NGS long-reads from two different chimpanzee individuals sequenced for this study (Chaos and Toby). The datasets are publicly available in NCBI SRA repositories under the accession number PRJNA905805 (Methods). Our analyses of the WGS data confirmed the computed subtelomeric structures, and found no significant polymorphisms (data not shown), further confirming the structure of the obtained assemblies.

## Discussion

We have shown the extent to which PhaseDancer can serve as an efficient, robust, and reliable tool resolving complex SD-rich genomic regions. Compared to the latest, commonly used assemblers, it provides the most accurate data, even for SDs with highly complex structures in the shortest time. Moreover, such tasks are accomplished also for the error-prone long reads.

Consequently, PhaseDancer has enabled substantial and robust extensions of the Great Apes subtelomeric regions evolutionarily important for the HSA2 formation. We have provided the validated and publicly available tool relying on the currently most efficient software and technologies that can be further developed and extended also at the community-based level.

**Fig. 5:**
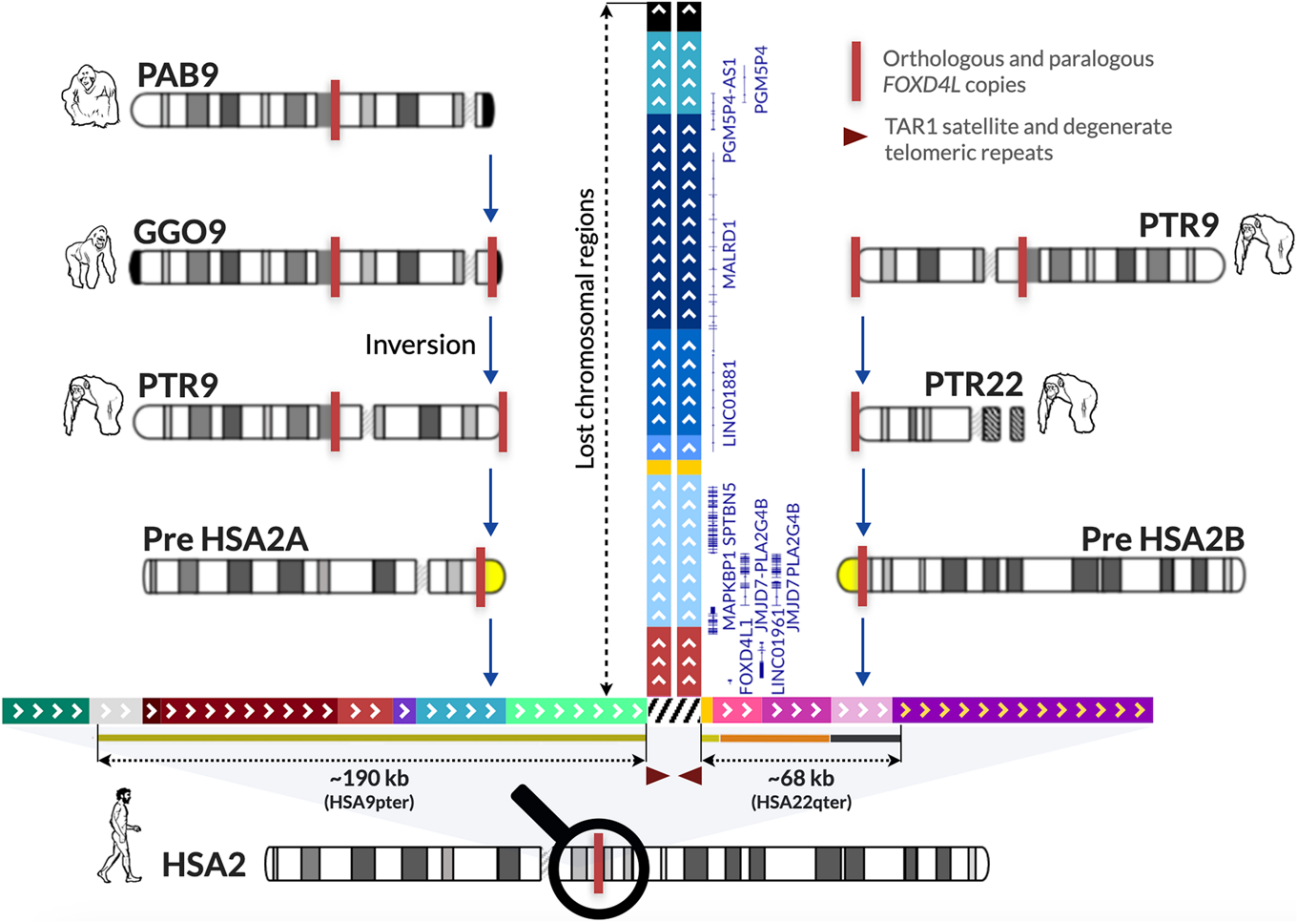
The proposed model for the evolutionary HSA2 fusion event based on the assembled SD-rich subtelomeric sequences in Great Apes chromosomes, absent in the reference genomes. The fusion site is flanked proximally and distally, respectively, by the ~190 kb and ~68 kb SDs homologous to human chromosomes 9p24.3 and 22q13.33 (98.9% and 97.8-99.1% sequence identity). The ~190 kb fragment harbouring *FOXD4L1* (red solid rectangle) (Fig. 4), and likely originating from an ancestral locus syntenic to chromosome 9q21.11 in human, was previously shown to be duplicatively transposed to chromosome PTR2Apter after gorilla had branched off the common chimp-human ancestor lineage (Additional file 1: Table S3-S5) [20, 36, 46, 47]. Both copies flank the evolutionarily pericentromeric inversion in the human and chimp genomes that arose after the gorilla divergence [36, 45, 47]. We have proposed that a portion of the PTR9pter copy was also copied onto chromosome PTR22qter and later PTR2Bter before the gorilla-chimp divergence [36, 45, 48, 49]. Importantly, our assemblies revealed substantially long homology (~190kb) between the lost fragments (within the yellow band) of the ancestral chromosomes 2Apter (Pre HSA2A) and 2Bpter (Pre HSA2B) that might have served as a substrate of misalignment during meiosis. The fusion occurred within TAR1 satellite and degenerate telomeric repeats present in both Pre HSA2Apter and Pre HSA2Bpter. Submicroscopic subtelomeric rearrangements in human are relatively common cause of genomic imbalances in patients with developments delay/intellectual disability [50]. Analyses of these sequences showed that two copies of the following six protein coding genes *FOXD4L1*, *JMJD7-PLA2G4B*, *MAPKBP1*, *SPTBN5*, *CBWD2*, and *MALRD1*, one pseudogene *PGM5P4*, and three lncRNAs *LINC01881*, *LINC01961*, and *PGM5P4-AS1* might have been lost during the fusion event (Fig. 4, Additional file 1: Fig. S9)

The results of our assemblies have allowed us to propose a scenario of the evolutionary formation of the HSA2 fusion involving not only chromosomes 2Apter and 2Bpter as hypothesised in the current models, but also 9pter and 22qter chromosomes (Fig. 5). The existing reference genome sequences of the SD- and StSat-rich subtelomeric regions in the majority of Great Apes chromosomes remain, to a large extent, incomplete. Corroboratively, our assembled sequences of chromosomes 2Apter and 2Bpter in chimp and gorilla are in concordance with the previously published results of the FISH studies with the human cosmid and fosmid probes from the HSA2 fusion site [20, 45].

Supporting the notion of Ventura et al. [20] that the pericentric inversion of chromosome 2A predisposed the chimpanzee and human genomes to formation of StSat-rich subtelomeric heterochromatin, whereas the HSA2 fusion prevented our genome from these expansions, we found multiple copies of two unstable genomic segments admixed with the StSat repetitive DNA sequences on the subtelomeric regions of chromosomes 2Apter and 2Bpter in chimpanzee and bonobo as well as on chromosome 2Bpter in gorilla. The copies of the above-mentioned ~31 kb fragment mapping proximally to the fusion site and amplified ~400 times in the chimp genome [19] are admixed to StSat sequences on chromosomes 2Bpter in chimp and 2Apter in bonobo (Figs. 2D, 4, 5). Moreover, the copies of the ~82 kb block 1 (chr10:19,112,612-19,194,164) and the ~43 kb block 2 (chr10:19,238,586-19,281,823) [4, 20], originating from the ancestral locus orthologous to HSA 10p12.31 and expanded in gorilla with greater than 100 copies and 23-50 copies in chimpanzee and bonobo, but present only in a single copy in human (Additional file 1: Fig. S10), are directly admixed to StSat sequences on chromosome 2Bpter both in bonobo (chr10:19,112,645-19,123,078) and in gorilla (chr10:19,220,718-19,229,071 and chr10:19,233,190-19,244,822) as proposed by Ventura et al. [20].

Out of the six protein coding genes (each in two copies) *FOXD4L1*, *JMJD7-PLA2G4B*, *MAPKBP1*, *SPTBN5*, *CBWD2*, and *MALRD1,* one pseudogene *PGM5P4*, and three lncRNAs *LINC01881*, *LINC01961*, and *PGM5P4-AS1* that might have been deleted during the fusion event (Figs. 4, 5), thus far, only *MAPKBP1* has been disease-related in human in an autosomal recessive manner (MIM 617271). Interestingly, *FOXD4*, a member of the forkhead/winged helix-box transcription factor gene family, highly conserved among vertebrates, has been shown recently to play an important role in brain development. In *Xenopus* embryo, *Foxd4l1.1* (previously *Foxd5a/b*), known to play an essential role in maintaining an immature neural fate by regulating several neural transcription factors [51, 52], was found to strongly inhibit mesoderm- and ectoderm-specific marker genes to maintain neural fate by negatively regulating Chordin transcription [53]. In mice, *Foxd4*, required in the transition of the mouse embryonic stem cells from pluripotency to neuroectoderm precursor cells, was found to be essential in the anterior mesoderm and in the anterior neuroectoderm for rostral neural tube closure and neural crest specification during head development. Interestingly, loss of *Foxd4* manifested with craniofacial malformations and neural tube closure defects [54]. *Foxd4* in mice is also essential for establishing neural cell fate and for neuronal differentiation [55]. Loss of *FOXD4* in human was proposed to be responsible for developmental delay in patients with Chromosome 9p deletion (9p-) syndrome (MIM 158170) [56]. However, the *FOXD4* gene paralogs have not been disease associated, likely because of their multi-copy redundancy. Of note, we found increased expression of all human *FOXD4* paralogs in cerebellum and *FOXD4L2* in tibial nerve (https://gtexportal.org/), suggesting their potential role in bipedalism.

HSA2 was estimated to have occurred 0.74 Mya [57], ~3.5 Mya [37], greater than 4 Mya [20], between 1-6 Mya [45], and between 5-6 Mya [38]. Most recently, by re-analysing the enrichment of weak-to-strong (AT to GC) substitutions around the fusion site, we dated its formation at ~0.9 Mya with an upper boundary of ~1.5 Mya [58]. However, it is tempting to speculate that HSA2 fusion was a major evolutionarily event that had initiated the separation of *Hominina* from *Pan* (chimpanzee and bonobo) and introduced the reproductive barrier between them. Moreover, the early HSA2 stabilisation by fusion of chromosomes 2A and 2B harbouring these genome destabilising chr2 and chr10 segments could explain the absence of the StSat-rich cap sequences (StSat, SatIII, and rDNA ) expanded in gorilla, chimpanzee, and bonobo [20]. Our genomic analyses in 13 individuals revealed no evidence of variability at the HSA2 fusion site, including the terminal degenerate repeats as well as the flanking complex SDs in humans (Additional file 1: Fig. S8, Tables S1, S2), implying that HSA2 fusion was most likely a nonrecurrent event. We have proposed that large paralogous sequences on distal chromosomes 2Apter and 2Bpter, representing, respectively, orthologous regions on chromosomes 9pter and 22qter in Great Apes, might have facilitated meiotic misalignment between these chromosomes. Our computational analyses of the Great Apes genomes revealed that the ~800 bp TAR1 satellite and degenerate telomeric repeats present at the HSA2 junction site have orthologous copies in both PTR2Apter and PTR2Bpter, indicating where the break and fusion might have occurred (Fig. 5).

## Conclusions

PhaseDancer is a cutting-edge tool for targeted de-novo genomic assemblies, including complex SD-rich regions. The potential applications include also: (i) assembly of the subtelomeric and complex regions of human chromosomes, (ii) fast assembly of the unique genomic regions, and (iii) assessment of the SD copy-number. In addition to the presented evolutionary events it also has potential in personalised medicine for targeting patient-specific SD-related disorders.

## Methods

### Datasets

#### Whole genome sequencing of two chimpanzees

Using long-read PacBio Sequel II technology, we whole genome sequenced two chimpanzee (Chaos and Toby from the Houston Zoo) genomes. Chaos’ genome was sequenced using CLR technology with 70x coverage, whereas Toby’s genome using CCS (HiFi) technology with 20x coverage [59].

First, the peripheral blood DNA samples were assessed as suitable for PacBio Sequel II sequencing. DNA was fragmented with the Covaris^®^ g-TUBE^®^ device. Next, DNA damages were repaired using the DNA Damage Repair reagents (PacBio). To ligate the hairpins (SMRTbell™ templates) to the DNA fragments, BLUNT hairpin adapters (20$\mu$M) oligonucleotide pre-annealed stocks) were used. To remove failed ligation products, exonuclease was added. Three-step AMPure PB Size-Selection and Purification was performed. Prior to sequencing, primer was annealed to both ends of the SMRTbell template. The binding reaction was performed and DNA sequencing polymerases were bound to the primer-annealed SMRTbell templates (at 30°C for 30 minutes). The template-polymerase complex was transferred to a 96-well sample plate with adjusted concentrations and volumes. The DNA fragments in a zero-mode waveguide well were sequenced using PacBio Sequel II repeatedly in the sequencing process. The obtained broadcasts were self corrected to obtain highly accurate CCS reads. The resulting CCS data quality control confirmed its validity to perform the downstream analyses of the WGS from PacBio Sequel II. The P1 ratio of the two cells was over 89.62%, the average length of subreads was 14,666 bp, the read N50 was 22,239 bp, the longest read length is 268,467 bp, and the total data was 231,859,915,436 bp.

#### Reference genomes

All reference genomes of human and Great Apes used in this study were downloaded from the UCSC Genome Browser (https://hgdownload.soe.ucsc.edu/downloads.html) [60]:

- Genome Reference Consortium Human GRCh38.p13; hg38 assembly of human genome (December 2013);
- T2T Consortium/T2T-CHM13 v2.0 assembly of the human genome (January 2022);
- University of Washington Clint_PTRv2; panTro6 assembly of the chimpanzee (*Pan troglodytes*) genome (University of Washington, January 2018);
- Chimpanzee Sequencing and Analysis Consortium Build 3.0; panTro5 assembly of the chimpanzee (*Pan troglodytes*) genome (May 2016);
- University of Washington Mhudiblu_PPA_v0 assembly; panPan3 assembly of the the bonobo (*Pan paniscus*) genome (May 2020);
- Max-Planck Institute for Evolutionary Anthropology panpan1.1; panPan2 assembly of the bonobo (*Pan paniscus*) genome (August 2015);
- University of Washington Kamilah_GGO_v0; gorGor6 assembly of the gorilla (*Gorilla Gorilla*) genome (August 2019);
- University of Washington Susie_PABv2; ponAbe3 assembly of the orangutan (*Pongo pygmaeus abelii*) genome (University of Washington, January 2018).

#### Great Apes NGS data from public repositories

The following PacBio circular consensus sequencing (CCS) data for Great Apes were used to validate and extend the existing references:

- Chimpanzee (Clint), BioSample SAMN15896587, Bioproject PRJNA659034 (Primate genome sequencing and assembly) [61],
- Bonobo (Mhudiblu), BioSample SAMN11123633, Bioproject PRJNA691628 (bonobo and gorilla HiFi reads) [62],
- Gorilla (Kamilah), BioSample SAMN11078986, Bioproject PRJNA691628 (bonobo and gorilla HiFi reads) [62],
- Orangutan (Susie), BioSample SAMN15896588, Bioproject PRJNA659034 (Primate genome sequencing and assembly) [61].

#### Analysis of polymorphisms

To assess the polymorphisms flanking the HSA2 fusion site, we analysed NGS data (Nanopore, PacBio CLR, and CCS HiFi) from two data sources: Genome in the Bottle (3 individuals [63–65]) and T2T Diversity Panel (10 individuals: HG01109, HG01243, HG02080, HG03098, HG02055, HG03492, HG02723, HG02109, HG01442, HG02145 [66]).

#### Optical genome mapping data

All OGM data representing assembly of raw molecules in CMAP format were provided by Bionano Genomics and downloaded from NCBI FTP sites (see the Availability of data and materials section). We used the optical genomic maps generated with the nicking enzymes BssSI and BspQI of the chimpanzee and orangutan genomes from the bioproject PRJNA369439 [67], and the bonobo genome from the bioproject PRJNA672266 [68]. Gorilla Bionano Genomics data from the bioproject PRJNA369439 [67] were generated with DLE-1 enzyme.

#### Transcriptomic analysis

Bulk RNA-seq data from three species: human, bonobo, and chimpanzee available at Sequence Read Archive under the bioproject PRJNA527986 [69] were used to perform the comparative analyses of the transcriptomes from the PhaseDancer-extended subtelomeric regions.

### PhaseDancer - workflow details

PhaseDancer uses an iterative greedy strategy for repetitively extending the short initial anchor sequence by executing the following phases: (i) mapping, (ii) clustering, (iii) assembling, and (iv) extending (Fig. 1). Additionally, we have described in details the accompanying tools: PhaseDancerViewer and PhaseDancerSimulator.

To start working with PhaseDancer, a pipeline is used to: (i) build an index based on the read data (using minimap2 [10] tool), (ii) load the index to the RAM, and (iii) prepare the initial anchor sequences to be extended by PhaseDancer.

### Mapping phase

An anchor sequence is mapped on the set of all reads using an inverted index loaded to RAM. Some randomly selected reads from the sample are then sent to the standard input of the mapper to load the buffer of minimap2, forcing the tool to output mappings at least once per iteration. As a result, the process of receiving the output from the mapper determines the end of the entries from the anchor sequence. The output from the anchor sequence is further processed when the first entry from the randomly selected reads is recognised.

The Pairwise mApping Format (PAF) entries generated by minimap2 are then processed to filter the reads with sufficiently large coverage (parameterised by default with at least half of the anchor length). Selected reads are then retrieved from a FASTA file using the Faidx index.

Finally, the reads are homopolymer-compressed (HPC) and mapped on the HPC anchor sequence to produce a BAM file that is an input to the next PhaseDancer phase.

### Clustering phase

The HPC reads overlapping the full HPC anchor sequence are selected using the BAM alignment file from the previous step. Using this alignment mismatches are analysed to find candidates for cis-morphisms. Here, a cis-morphism refers to a single nucleotide difference between two or more segmental duplications.

To detect cis-morphisms, the frequency of the second most common nucleotide is computed for each locus. A locus is identified as a cis-morphism when the corresponding second most common nucleotide frequency is greater than a given threshold value (parameter dependent on sequencing technology and the coverage). Additionally, when the number of the identified cis-morphisms is greater than a given upper-bound (by default set to 200), only those with the largest percent of the second most common base are retained. Such filtered cis-morphisms are then used for clustering.

The first step of clustering is based on the graph connectivity analysis. A graph used for clustering reads is called a similarity graph. A set of vertices of the similarity graph corresponds to the reads overlapping the full HPC anchor sequence. Each edge of the similarity graph connects vertices most similar to each other according to a Hamming distance of cis-morphisms (0.4). The decomposition of the similarity graph into the connected components corresponds to the partition of the computed reads.

In the second step, each block in the partition is subdivided into clusters using cis-morphisms derived from the reads composing the block. The clustering process is based on random simulations and generates multiple alternative clusterings of reads.

In each simulation, a random cis-morphism is selected iteratively to partition the set of all reads based on the observed nucleotides. The procedure is applied recursively until either no cis-morphisms are present in the processed set of reads, or the number of reads in each constructed cluster falls below a certain threshold (this threshold is sequencing technology-dependent, yet it is assumed to be 0.8 $\times$ coverage).

Given all the alternative clusterings of reads, we assign the best clustering to each block. To evaluate the quality of a clustering, we computed the sum of distances between each read and its nearest cluster. In this context, the distance between a read and a cluster is determined by the Hamming distance between the read and the consensus sequence derived from all reads in the cluster. The best clustering is the one that minimises the sum of distances across all reads.

The final clustering of all reads is a union of all clusters from all blocks. The cluster used for the extension of the anchor sequence maximises the number of reads shared with the cluster selected for the extension in the previous iteration. In particular, in the first iteration, the cluster is selected by the similarity to the initial sequence (i.e. Hamming distance between the sequence and clusters consensuses).

### Assembling phase

Reads from the selected cluster are pre-processed based on their mapping to the anchor sequence by truncating fragments exceeding the sequence by given flanking threshold. The procedure is applied to ensure even coverage and the fixed length of the assembly required by wtdbg2 [13]. Then, the reads are assembled using this tool. This process is fast and precise as it operates only on the reads from one cluster originating from one genomic region with the read number approximated by the coverage of the sequencing data.

### Extending phase

The newly assembled sequence is aligned to the anchor sequence using the edlib library [70] minimising the Levenshtein distance. The flanking part is used for the extension of the current anchor sequence to the new anchor sequence processed in the next iteration (Mapping phase) of PhaseDancer.

### Implementation details

PhaseDancer was implemented as a Snakemake [71] workflow. The source code, the docker image of PhaseDancer, and the toy-example along with the detailed manual are available online [72, 73].

PhaseDancer uses the index of all sequencing data loaded into RAM to query for reads that are similar to the anchor sequence. Therefore, before running the main workflow the index build for all sequencing data needs to be generated. PhaseDancer uses minimap2 .mmi files generated with:

–idx-no-seq parameter to reduce the memory required for the index to be stored (if used, the mapper can produce an output only in the PAF format),
-p 0 -N 3000 parameters to ensure that all reads having fragments similar to the anchor sequence are outputted,
-K 1 parameter to force the mapper to generate an output once per read.

As a reference point for the memory usage, an index of 200 GB stored in a FASTA file uses approximately 150 GB of RAM.

Before the first iteration of PhaseDancer, the minimap2 index has to be loaded into the RAM together with two processes running in an infinite loop and handling the standard input and output of the mapper. The former receives sequences from the pipeline and sends them to the standard input of the mapper, the latter receives the output in the PAF format from the mapper, selects the reads using the Faidx index, and sends them back to the pipeline.

PhaseDancer enables the concurrent extension of many sequences. To accomplish this functionality, the input sequences are sent to the mapper using the flock command. Then, the process retrieving the mapping results allows for the multiplexing of PAF entries sent from many other processes. Distinction of the sender process of an entry is based on a uniquely identifying name.

### PhaseDancerViewer - intermediate results viewer

PhaseDancer is accompanied by PhaseDancerViewer, an application for the visualisation of its intermediate assembly results obtained at the end of each algorithm iteration. The viewer enables monitoring the assembly process in a semi-supervised mode. User can interfere the assembly process and re-tune the parameters of PhaseDancer. For every iteration, it displays the reads mapped on an anchor sequence grouped and colored by clusters. The application visualizes clusters using an embeddable implementation of the Integrative Genomics Viewer (IGV). The source code with the documentation is available online [74, 75].

### PhaseDancerSimulator - SDs generator

PhaseDancer is targeted at resolving SD-rich genomic regions, thus the standard methods dedicated to assemblers evaluation and benchmarking are unsuitable or even inadequate. To show the advantages of PhaseDancer and verify its robustness, we implemented a simulator generating contigs and recapitulating the complex history of SDs formation.

PhaseDanceSimulator extends the method proposed by Chaisson et al. [76]. The simulation process follows the simplified model based on the tree topology. Fragments from a reference genome are assigned to the root of the tree and child sequences are generated by copying a parent node sequence and mutating each base at a fixed rate per base. PhaseDancerSimulator supports four topology types: flat, bifurcating, cascading, and random (Tab. 1). Moreover, the ends of the generated contig sequences can be extended with a randomly generated sequence.

PhaseDancerSimulator supports Oxford Nanopore and PacBio Sequel technologies using PBSIM2 [77] to simulate reads. Other simulation parameters include, e.g. mutation rate, mean and standard deviation of the read length, read accuracy, chemistry, and coverage. Additionally, the tool can generate assemblies using Canu [11], Wtdbg2 [13], Flye [12], and Miniasm [10] that can be used for benchmarking of the assemblies.

The source code and the documentation of PhaseDancerSimulator are available online [78, 79].

### Runtime experiments

T2T data of SDs were used as a reference point to asses the distribution of the number of stacked SDs in the human genome needed to specify the parameters for the runtime experiments. We calculated the percent of all SD bases that have no more than *n* stacked SDs as: $\le 5 \approx 65\%$; $\le 10 \approx 79\%$; $\le 15 \approx 83\%$; $\le 20 \approx 87\%$; $\le 30 \approx 90\%$. Moreover, the median number of the stacked SDs for the interstitial SDs was equal to 2. Importantly, the cases with more than 20 stacked SDs related to very short fragments.

Therefore, to conduct runtime experiments, we generated data using PhaseDancerSimulator for the number of clusters varying from 1 up to 40 (mutation rate 0.001, P6C4 PacBio chemistry, coverage 40x, sequencing error 15%, mean read length 18 kb, read length standard deviation of 3 kb, flat tree topology). The upper bound was set to 40 because in the real data scenario cases with more clusters are extremely rare, thus they do not influence the effective runtime of the algorithm.

Importantly, when assessing the runtime of the PhaseDancer number, we observed that the main bottleneck of the PhaseDancer workflow is the clustering procedure. To optimise this step, we paralleled this procedure and measured the execution time of one iteration given the number of processes used.

For such generated datasets and the number of processes used (1, 5, 10, 20), we ran the experiments for 100 iterations aiming to assemble the ~ 0.5 Mb regions. To asses the time performance of PhaseDancer, one iteration time was computed for each run. The final results of the time experiments are presented in Fig. 3A.

### Optical genome mapping validation of the recent reference genomes and PhaseDancer assemblies in Great Apes

To validate the assemblies of the reference genomes used in our work, we used the Bionano Genomics data described above. Data processing pipeline followed the producer’s *Guidelines for Running Bionano Solve in the Command Line* (Guidelines at https://bionanogenomics.com/).

FASTA files of the genome reference builds were in silico digested with the nicking enzymes using HybridScaffold script to produce files in the CMAP format. Then, the mapping was performed using the producer provided runCharacterize.py script with preset parameters optArguments_haplotype_saphyr.xml (for BssSI and BspQI enzymes) and optArguments_haplotype_DLE1_saphyr.xml (for DLE-1 enzyme) accompanying the script. The produced mapping was visualised using the Bionano Access Server (Additional file 1: Figs. S2-S5).

### Bulk RNA-seq gene expression analysis

RNA-seq data [42] from 33 brain sites of human, chimpanzee, and bonobo were mapped on the masked reference human genome hg38 using the minimap2 [10]. The hard-masked sequences correspond to the fusion site syntenic regions. Hard-masking was done in order to force unique mapping of the transcripts on the near fusion site region.

A subset of transcripts that were identified on the PhaseDancer assembled subtelomeric sequence extensions: *CBWD2*, *FOXD4L1*, *JMJD7*, *JMJD7-PLA2G4B*, *LINC01881*, *LINC01961*, *MALRD1*, *MAPKBP1*, *PLA2G4B*, *RABL2A*, and *SPTBN5* was selected to perform the downstream comparative transcriptomic analysis. The selected transcripts coordinates at hg38 genome were downloaded using UCSC hgTables form GENECODE V41 track.

The downstream analysis was performed using a custom-made python script. The analysis starts by defining for each transcript the set of coordinates that describe any of its exomes. For each coordinate, we calculated its coverage using the pileup query. Next, for each transcript (for all its exome coordinates) we calculated the average coverage normalised by the sample size (i.e. the total length of all reads in the brain region RNA-seq data in question). The final results were visualised and compared between the brain regions using R-script (Additional file 1: Fig. S9).

### Supplementary Information

**Additional file 1: Table S1.** RepeatMasker analysis of HSA2 fusion site flanking regions of 3 human genomes from Genome in the Bottle project repository. **Table S2.** RepeatMasker analysis of HSA2 fusion site flanking regions of 10 human genomes from T2T Diversity Panel. **Table S3.**[XMLSPACE]*FOXD4* gene family in the human hg38 genome build. **Table S4.** Segmental duplications harbouring *FOXD4* gene paralogs in human in the hg38 human genome build. **Table S5.** Listing of *FOXD4* gene family orthologs locations gene in Great Apes. **Figure S1.** Gross inversion events in the course of primate evolution. **Figure S2.** Assessment of the current orangutan reference genome quality (ponAbe3) using Bionano Genomics with nicking enzymes BssSI and BspQI. **Figure S3.** Assessment of the current gorilla reference genome quality (gorGor6) using Bionano Genomics, enzyme DLE-1. **Figure S4.** Assessment of the current chimpanzee reference genomes quality (panTro5 and panTro6) using Bionano Genomics. **Figure S5.** Assessment of the current bonobo reference genomes quality (panPan2 and panPan3) using Bionano Genomics. **Figure S6.** Example of the GorGor6 reference genome assembly error. **Figure S7.** Comparison of the q arm of chromosome 2B in PanTro5 and PanTro6. **Figure S8.** Multialignment of the genomic fragments flanking the HSA2 fusion site. **Figure S9.** Expression levels of 11 transcripts in chimpanzee, bonobo, and human (*CBWD2*, *FOXD4L1*, *JMJD7*, *JMJD7-PLA2G4B*, *LINC01881*, *LINC01961*, *MALRD1*, *MAPKBP1*, *PLA2G4B*, *RABL2A*, and *SPTBN5*) found on the extensions of the subtelomeric regions assembled with PhaseDancer. **Figure S10.** Normalised depth-of-coverage histogram of the aligned whole-genome CCS reads of a 225-kbp region of human chromosome 10 (chr10:19075000-19300000, NCBI hg38) in human (NA12878), two chimpanzees (Clint, Chaos), bonobo (Mhudilbu) and gorilla (Kamilah).

**Additional file 2.** Review history.

## Data Availability

The sequence data (.FASTQ files) of Toby and Chaos chimpanzees generated for this study are available in the National Center of Biotechnology Information (NCBI) under the Sequence Read Archive (SRA) as BioProject No. PRJNA905805 with BioSample IDs SAMN31883988 and SAMN31883989, respectively, and SRA accession number PRJNA905805 [59]. Publicly available datasets from the Sequence Read Archive (SRA) used in this study: $\bullet$ Pacbio CCS reads of Chimpanzee (Clint), BioSample SAMN15896587, Bioproject PRJNA659034 [61]. $\bullet$ Pacbio CCS reads of Bonobo (Mhudiblu), BioSample SAMN11123633, Bioproject PRJNA691628 [62]. $\bullet$ Pacbio CCS reads of Gorilla (Kamilah), BioSample SAMN11078986, Bioproject PRJNA691628 [62]. $\bullet$ Pacbio CCS reads of Orangutan (Susie), BioSample SAMN15896588, Bioproject PRJNA659034 [61]. $\bullet$ RNA-seq data from three species: human, bonobo, and chimpanzee, Bioproject PRJNA527986 [69]. $\bullet$ Bionano CMAP data of Chimpanzee, Biosample SAMN06272697, Bioproject PRJNA369439 [67]. $\bullet$ Bionano CMAP data of Bonobo, Biosample SAMN16561985, Bioproject PRJNA672266 [68]. $\bullet$ Bionano CMAP data of Gorilla, Biosample SAMN11078986, Bioproject PRJNA369439 [67]. $\bullet$ Bionano CMAP data of Ornagutan, Biosample SAMN06275555, Bioproject PRJNA369439 [67]. The list of all OGM data hosted by NCBI was downloaded from NCBI FTP sites using URLs provided in the following link: ftp://ftp.ncbi.nlm.nih.gov/pub/supplementary_data/bionanomaps.csv. The study utilized publicly available datasets from the Genome in a Bottle project: $\bullet$ Pacbio CCS reads of Ashkenazim son (HG002) genome [63]. $\bullet$ Pacbio CCS reads of Chinese Trio son (HG005) genome [64]. $\bullet$ Pacbio CCS reads of female from Utah (NA12878:HG001) genome [65]. Publicly available Pacbio CCS reads of 10 human genomes from T2T Diversity Panel (HG01109, HG01243, HG02080, HG03098, HG02055 HG03492, HG02723, HG02109, HG01442, HG02145) were downloaded from AWS Web Hosting Services listed on the web page of T2T Diversity Panel [66]. All the source codes related to the article are available in GitHub repositories and associated with DOI via Zenodo: $\bullet$ https://github.com/bposzewiecka/phaseDancer [72] - DOI: 10.5281/zenodo.7948970 [73] $\bullet$ https://github.com/bposzewiecka/phaseDancerViewer [74] - DOI: 10.5281/zenodo.7948964 [75] $\bullet$ https://github.com/bposzewiecka/phaseDancerSimulator [78] - DOI: 10.5281/zenodo.7924985 [79] and are all distributed under the GNU General Public Licence version 2.
