# Supplementary material for: PhaseDancer: a novel targeted assembler of segmental duplications unravels the complexity of the human chromosome 2 fusion going from 48 to 46 chromosomes in hominin evolution
Source: Genome Biol. 2023 Sep 11;24:205. doi: 10.1186/s13059-023-03022-8 (PMC10496407; doi:10.1186/s13059-023-03022-8)
Supplement: Supplementary file 1 — Additional file 1: Table S1. RepeatMasker analysis of HSA2 fusion site flanking regions of 3 human genomes from Genome in the Bottle project repository. Table S2. RepeatMasker analysis of HSA2 fusion site flanking regions of 10 human genomes from T2T Diversity Panel. Table S3.[XMLSPACE]FOXD4 gene family in the human hg38 genome build. Table S4. Segmental duplications harbouring FOXD4 gene paralogs in human in the hg38 human genome build. Table S5. Listing of FOXD4 gene family orthologs locations gene in Great Apes. Figure S1. Gross inversion events in the course of primate evolution. Figure S2. Assessment of the current orangutan reference genome quality (ponAbe3) using Bionano Genomics with nicking enzymes BssSI and BspQI. Figure S3. Assessment of the current gorilla reference genome quality (gorGor6) using Bionano Genomics, enzyme DLE-1. Figure S4. Assessment of the current chimpanzee reference genomes quality (panTro5 and panTro6) using Bionano Genomics. Figure S5. Assessment of the current bonobo reference genomes quality (panPan2 and panPan3) using Bionano Genomics. Figure S6. Example of the GorGor6 reference genome assembly error. Figure S7. Comparison of the q arm of chromosome 2B in PanTro5 and PanTro6. Figure S8. Multialignment of the genomic fragments flanking the HSA2 fusion site. Figure S9. Expression levels of 11 transcripts in chimpanzee, bonobo, and human (CBWD2, FOXD4L1, JMJD7, JMJD7-PLA2G4B, LINC01881, LINC01961, MALRD1, MAPKBP1, PLA2G4B, RABL2A, and SPTBN5) found on the extensions of the subtelomeric regions assembled with PhaseDancer. Figure S10. Normalised depth-of-coverage histogram of the aligned whole-genome CCS reads of a 225-kbp region of human chromosome 10 (chr10:19075000-19300000, NCBI hg38) in human (NA12878), two chimpanzees (Clint, Chaos), bonobo (Mhudilbu) and gorilla (Kamilah). [file 13059_2023_3022_MOESM1_ESM.pdf]

PhaseDancer: a novel targeted assembler of segmental duplications unravels the complexity of the human chromosome 2 fusion going from 48 to 46 chromosomes in hominin evolution.

Supplementary Information

Barbara Poszewiecka, Krzysztof Gogolewski, Justyna A. Karolak, Paweł Stankiewicz and Anna Gambin

Table S1: **RepeatMasker analysis of HSA2 fusion site flanking regions of 3 human genomes from Genome in the Bottle project repository.** Contigs spanning the fusion site were assembled using PhaseDancer for 3 human genomes: HG001, HG002, HG005. Each of these contigs was aligned to the 5kb region directly flanking the HSA2 fusion site (chr2:113,601,000-113,605,999 (hg38)). The table lists the output of the RepeatMasker for the aligned fragments for the repeats immediately flanking the fusion site (TAR1 satellite, G-rich low-complexity region, (CTAACC)n simple repeat, and inverted TAR1 satellite).

score	% div.	% del.	% ins.	query begin	query end	strand	repeat	class/family	repeat begin	repeat end
Reference hg38										
2872	11,6	0,7	1,3	976	1681	+	TAR1	Satellite/subtelo	1	702
136	13,6	0,4	2,5	1683	1929	+	G-rich	Low_complexity	1	242
329	8,5	1,5	3,5	1930	2478	+	(CTAACC)n	Simple_repeat	1	538
5395	10,3	2,3	1,3	2479	3531	C	TAR1	Satellite/subtelo	(316)	1063
HG001										
2872	11,6	0,7	1,3	976	1681	+	TAR1	Satellite/subtelo	1	702
173	13,4	0,3	2,3	1683	1990	+	G-rich	Low_complexity	1	302
329	8,5	1,5	3,5	1991	2539	+	(CTAACC)n	Simple_repeat	1	538
5489	10,2	2,4	1,6	2540	3624	C	TAR1	Satellite/subtelo	(316)	1092
HG002										
2701	12	0,8	0,9	976	1652	+	TAR1	Satellite/subtelo	1	676
181	13,3	0,3	2,2	1654	1973	+	G-rich	Low_complexity	1	314
329	8,5	1,5	3,5	1974	2522	+	(CTAACC)n	Simple_repeat	1	538
5395	10,3	2,3	1,3	2523	3575	C	TAR1	Satellite/subtelo	(316)	1063
HG005										
2863	11,8	0,7	1,3	977	1682	+	TAR1	Satellite/subtelo	1	702
165	13,8	0,3	2,7	1684	1986	+	G-rich	Low_complexity	1	296
303	7,3	1,8	3,9	1987	2532	+	(CTAACC)n	Simple_repeat	1	535
5200	10,7	4,1	0,6	2533	3566	C	TAR1	Satellite/subtelo	(319)	1069

Table S2: **RepeatMasker analysis of HSA2 fusion site flanking regions of 10 human genomes from T2T Diversity Panel.** Contigs spanning the fusion site were assembled using PhaseDancer for 10 human genomes: HG01109, HG01243, HG02080, HG03098, HG02055, HG03492, HG02723, HG02109, HG01442, HG02145. Each of these contigs was aligned to the 5kb region directly flanking the HSA2 fusion site (chr2:113,601,000-113,605,999 (hg38)). The table lists the output of the RepeatMasker for the aligned fragments for the repeats immediately flanking the fusion site (TAR1 satellite, G-rich low-complexity region, (CTAACC)n simple repeat, and inverted TAR1 satellite).

score	% div.	% del.	% ins.	query begin	query end	strand	repeat	class/family	repeat begin	repeat end
Reference hg38										
2872	11,6	0,7	1,3	976	1681	+	TAR1	Satellite/subtelo	1	702
136	13,6	0,4	2,5	1683	1929	+	G-rich	Low_complexity	1	242
329	8,5	1,5	3,5	1930	2478	+	(CTAACC)n	Simple_repeat	1	538
5395	10,3	2,3	1,3	2479	3531	C	TAR1	Satellite/subtelo	(316)	1063
HG01109										
2872	11,6	0,7	1,3	976	1681	+	TAR1	Satellite/subtelo	1	702
181	13,3	0,3	2,2	1683	2002	+	G-rich	Low_complexity	1	314
325	8,6	1,5	3,4	2003	2544	+	(CTAACC)n	Simple_repeat	1	532
5395	10,3	2,3	1,3	2545	3597	C	TAR1	Satellite/subtelo	(316)	1063
HG01243										
2872	11,6	0,7	1,3	977	1682	+	TAR1	Satellite/subtelo	1	702
161	13,6	0,3	2,8	1684	1980	+	G-rich	Low_complexity	1	290
325	8,4	1,5	3,6	1981	2523	+	(CTAACC)n	Simple_repeat	1	532
5200	10,7	4,1	0,6	2524	3560	C	TAR1	Satellite/subtelo	(316)	1072
HG01442										
2872	11,6	0,7	1,3	976	1681	+	TAR1	Satellite/subtelo	1	702
176	13,6	0,3	2,3	1683	1996	+	G-rich	Low_complexity	1	308
329	8,5	1,5	3,5	1997	2545	+	(CTAACC)n	Simple_repeat	1	538
5489	10,2	2,4	1,6	2546	3630	C	TAR1	Satellite/subtelo	(316)	1092
HG02055										
2872	11,6	0,7	1,3	976	1681	+	TAR1	Satellite/subtelo	1	702
179	13,7	0,3	2,2	1683	2002	+	G-rich	Low_complexity	1	314
329	8,5	1,5	3,5	2003	2551	+	(CTAACC)n	Simple_repeat	1	538
5395	10,3	2,3	1,3	2552	3604	C	TAR1	Satellite/subtelo	(316)	1063
HG02080										
2872	11,6	0,7	1,3	976	1681	+	TAR1	Satellite/subtelo	1	702
180	12,8	0,3	2,3	1683	1996	+	G-rich	Low_complexity	1	308
329	8,5	1,5	3,5	1997	2545	+	(CTAACC)n	Simple_repeat	1	538
5473	10,3	2,4	1,6	2546	3630	C	TAR1	Satellite/subtelo	(316)	1092
HG02109										
2872	11,6	0,7	1,3	976	1681	+	TAR1	Satellite/subtelo	1	702
180	12,8	0,3	2,3	1683	1996	+	G-rich	Low_complexity	1	308
329	8,5	1,5	3,5	1997	2545	+	(CTAACC)n	Simple_repeat	1	538
5473	10,3	2,4	1,6	2546	3630	C	TAR1	Satellite/subtelo	(316)	1092
HG02145										
2872	11,6	0,7	1,3	977	1682	+	TAR1	Satellite/subtelo	1	702
165	13,8	0,3	2,7	1684	1986	+	G-rich	Low_complexity	1	296
322	8,3	1,5	3,6	1987	2523	+	(CTAACC)n	Simple_repeat	1	526
5195	10,9	2,4	1	2524	3545	C	TAR1	Satellite/subtelo	(316)	1036
HG02723										
2872	11,6	0,7	1,3	977	1682	+	TAR1	Satellite/subtelo	1	702
159	14,1	0,3	2,8	1684	1980	+	G-rich	Low_complexity	1	290
303	7,3	1,8	3,9	1981	2526	+	(CTAACC)n	Simple_repeat	1	535
5248	10,6	4,1	1,1	2527	3592	C	TAR1	Satellite/subtelo	(319)	1098
HG03098										
2872	11,6	0,7	1,3	977	1682	+	TAR1	Satellite/subtelo	1	702
161	13,6	0,3	2,8	1684	1980	+	G-rich	Low_complexity	1	290
325	8,4	1,5	3,6	1981	2523	+	(CTAACC)n	Simple_repeat	1	532
5200	10,7	4,1	0,6	2524	3560	C	TAR1	Satellite/subtelo	(316)	1072
HG03492										
2872	11,6	0,7	1,3	976	1681	+	TAR1	Satellite/subtelo	1	702
181	13,3	0,3	2,2	1683	2002	+	G-rich	Low_complexity	1	314
326	8,7	1,5	3,5	2003	2551	+	(CTAACC)n	Simple_repeat	1	538
5395	10,3	2,3	1,3	2552	3604	C	TAR1	Satellite/subtelo	(316)	1063

Table S3: ***FOXD4* gene family in the human hg38 genome build.**

Gene	Location	GRCh38/hg38	Size (bp)
FOXD4	9p24.3	chr9:116,231-118,417	2,187
FOXD4L1	2q14.1	chr2:113,498,665-113,501,150	2,486
FOXD4L3	9q21.11	chr9:68,302,867-68,305,084	2,218
FOXD4L4	9q21.11	chr9:65,737,146-65,738,396	1,251
FOXD4L5	9q21.11	chr9:65,282,101-65,285,209	3,109
FOXD4L6	9p11.2	chr9:41,126,435-41,128,445	2,011

Table S4: **Segmental duplications harbouring *FOXD4* gene paralogs in human in the hg38 human genome build.** The table presents the location, coordinates, size and the sequence identity (fracMatch) of these segmental duplications.

Gene	Location	GRCh38/hg38	Size (bp)	fracMatch (%)
FOXD4	9p24.3	chr9:10,412-203,762	193,351	-
FOXD4L1	2q14.1	chr2:113,413,433-113,602,734	189,302	98.85
FOXD4L3	9q21.11	chr9:68,220,553-68,322,179	191,627	98,29
FOXD4L4	9q21.11	chr9:65,653,950-65,738,783	84,834	98.45
FOXD4L5	9q21.11	chr9:65,282,740-65,325,123	42,384	98.37
FOXD4L6	9p11.2	chr9:41,109,162-41,211,432	102,271	98.22

Table S5: **Listing of *FOXD4* gene family orthologs locations gene in Great Apes.**

	Homo sapiens	Bonobo	Chimp	Gorilla	Orangutan
Build	GRCh38/hg38	Mhudiblu_PPA_v0/panPan3	Clint_PTRv2/panTro6	Kamilah_GGO_v0/gorGor6	Susie_PABv2/ponAbe3
Location	chr9q heterochromatin + fusion	chr9 interstitial + subtelomeric	chr9 interstitial + subtelomeric	chr9 interstitial + not subtelomeric	chr9 interstitial
FOXD4	chr9:116,231-118,417	chr9:109,355,827-109,358,069	chr9:110,485,476-110,487,705	chr9:39,427,431-39,429,069	chr9:41,142,975-41,145,933
FOXD4L1	chr2:113,498,665-113,501,150				
FOXD4L3	chr9:68,302,867-68,305,084				
FOXD4L4	chr9:65,737,146-65,738,396				
FOXD4L5	chr9:65,282,101-65,285,209				
FOXD4L6	chr9:41,126,435-41,128,445				
		chr2A:88,653,686-88,655,791			
		chr2B:569,315-571,435	chr2B:21,063-23,173		
				chr2B:18,030,229-18,030,865	
		chr2B:129,275,248-129,277,210	chr2B:128,729,228-128,731,369		
		chr16:70,838,835-70,841,084	chr16:75,871,836-75,874,281		
		chr18:74,033,352-74,035,603	chr18:74,730,904-74,733,523		
			chr3:41,729-43,926		
			chr9:58,241,637-58,243,881		
			chr22:33,666,462-33,668,678		
				chr14:26,299,807-26,300,445	
				chr16:28,193,811-29,770,630	

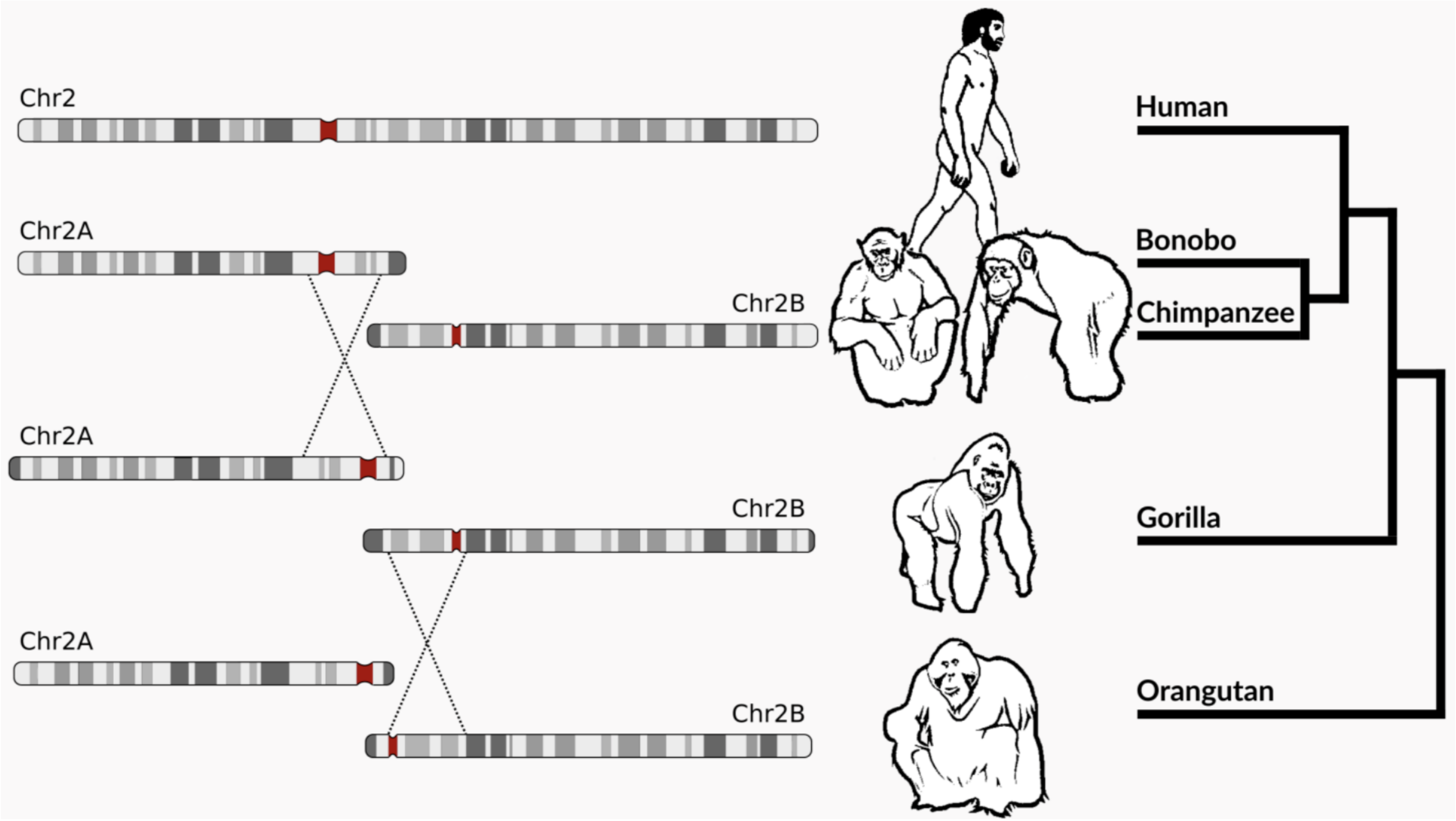

Figure S1: **Gross inversion events in the course of primate evolution.** Note that the orangutan's acrocentric Chr2B was inverted to form the gorilla's Chr2B, and later the chimpanzee and bonobo. Later, a similar event formed the chimpanzee and bonobo metacentric Chr2A after inversion on the gorilla acrocentric Chr2A. Eventually, the chromosomal fusion created the human Chr2 from the ancestral Chr2A and Chr2B and reduced the number of human chromosomes from 48 to 46.

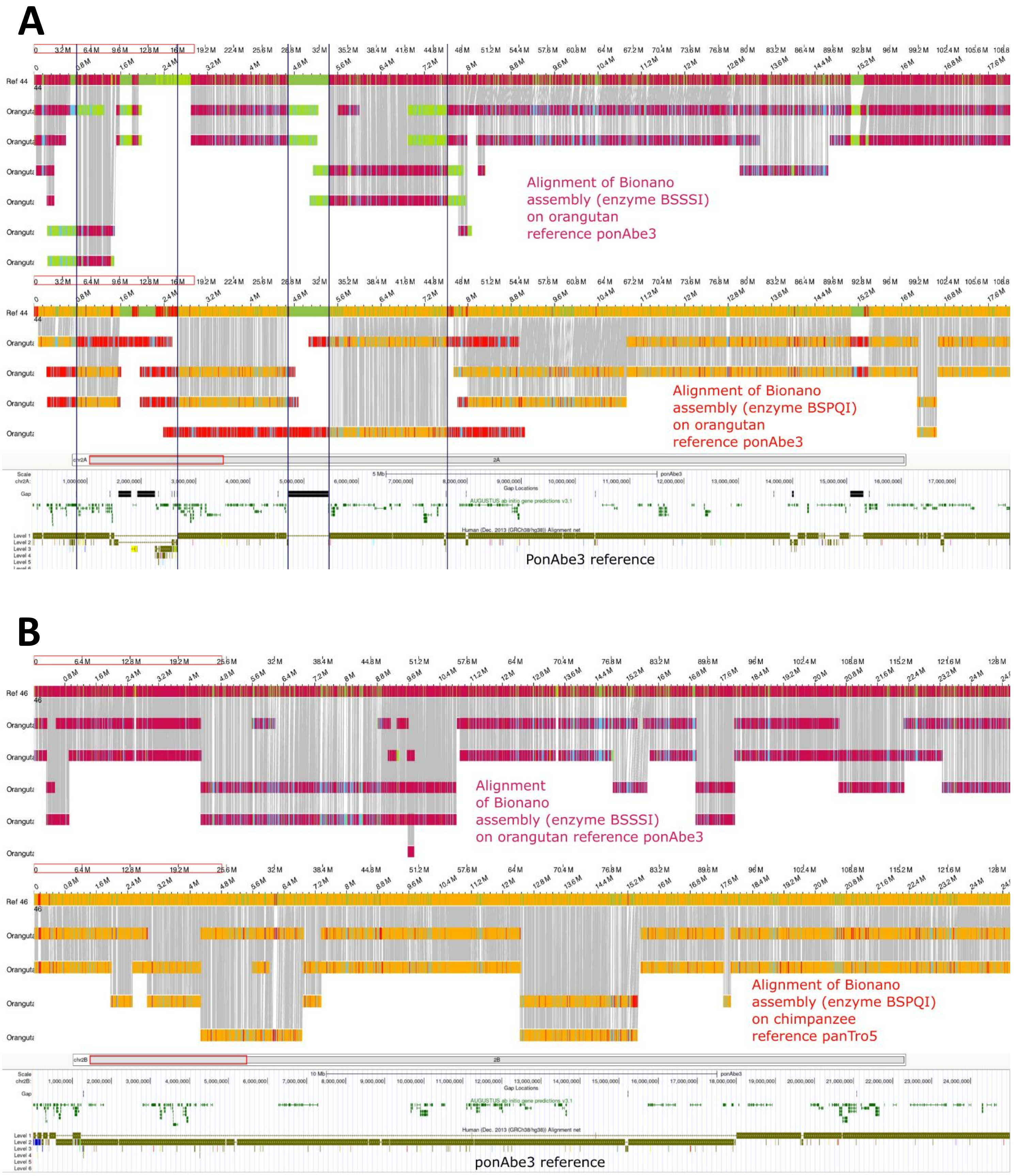

Figure S2: **Assessment of the current orangutan reference genome quality (ponAbe3) using Bionano Genomics with niking enzymes BSSSI and BSPQI.** (**A**) chromosome 2APTR; (**B**) chromosome 2BPTR.

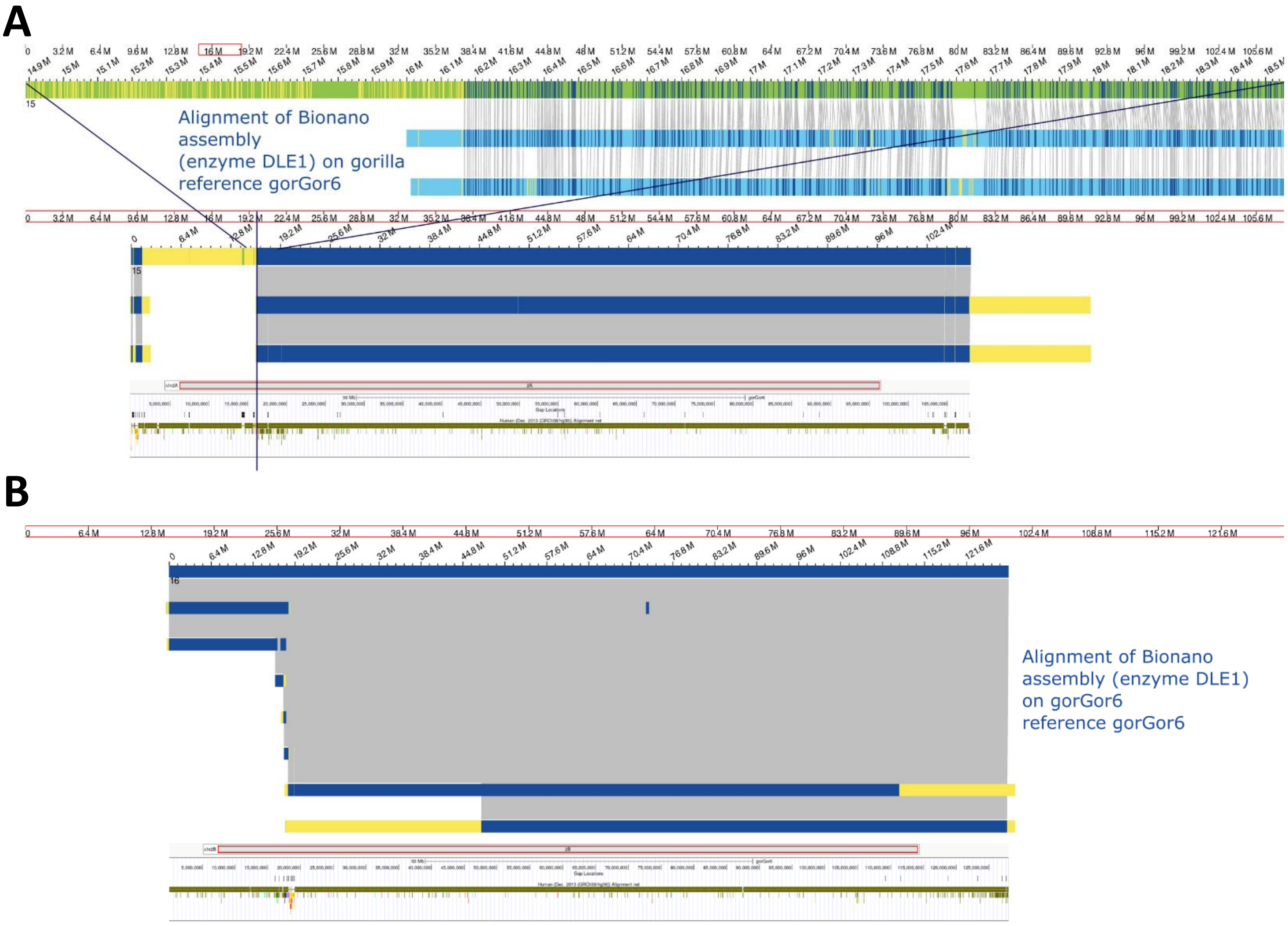

Figure S3: **Assessment of the current gorilla reference genome quality (gorGor6) using Bionano Genomics, nicking enzyme DLE1.** (**A**) chromosome 2APTR; (**B**) chromosome 2BPTR.

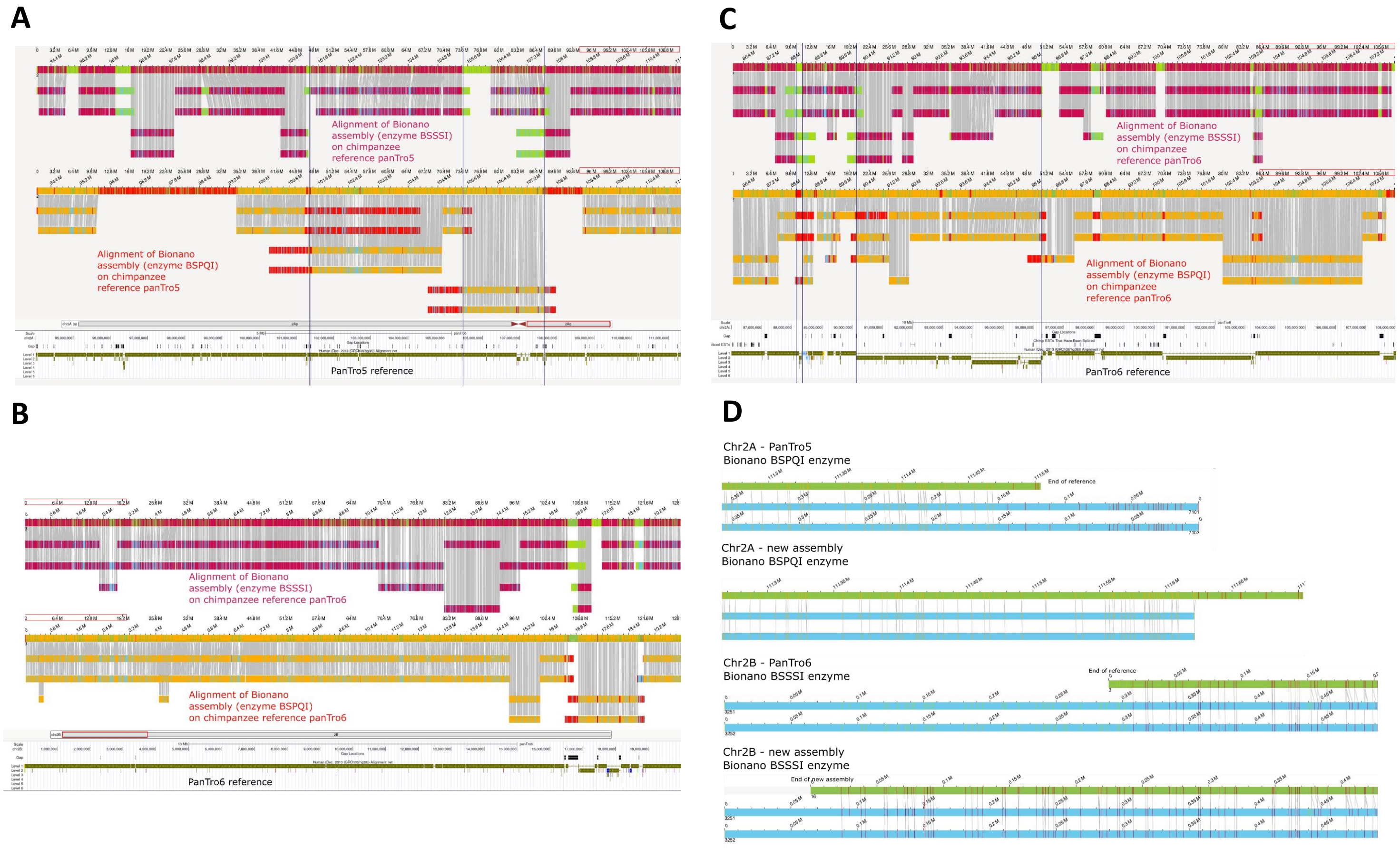

Figure S4: **Assessment of the current chimpanzee reference genomes quality (panTro5 and panTro6) using Bionano Genomics.** (**A**) chromosome 2APTR on panTro5 with BSSSI and BSPQI niking enzymes; (**B**) chromosome 2BPTR on panTro6 with BSSSI and BSPQI niking enzymes; (**C**) chromosome 2APTR on panTro6 with BSSSI and BSPQI niking enzymes; (**D**) comparison of the new assemblies generated by PhaseDancer with the current reference genomes.

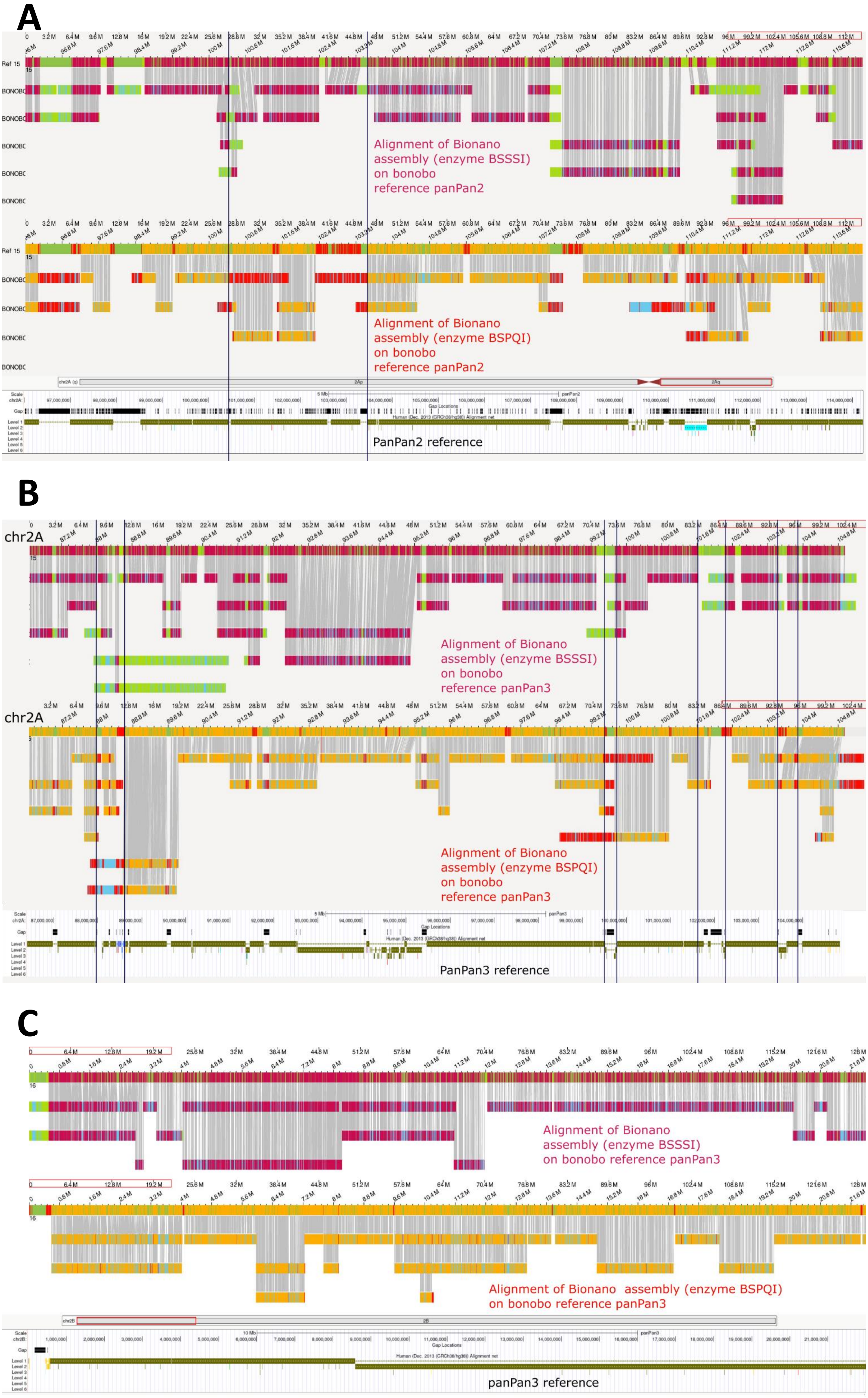

Figure S5: **Asf the q arm of chromosome 2B in PanTro5omes quality (panPan2 and panPan3) using Bionano Genomics.** (**A**) chromosome 2A on panPan2 with BSSSI and BSPQI niking enzymes; (**B**) chromosome 2A on panPan3 with BSSSI and BSPQI niking enzymes; (**C**) chromosome 2B on panTro3 with BSSSI and BSPQI niking enzymes.

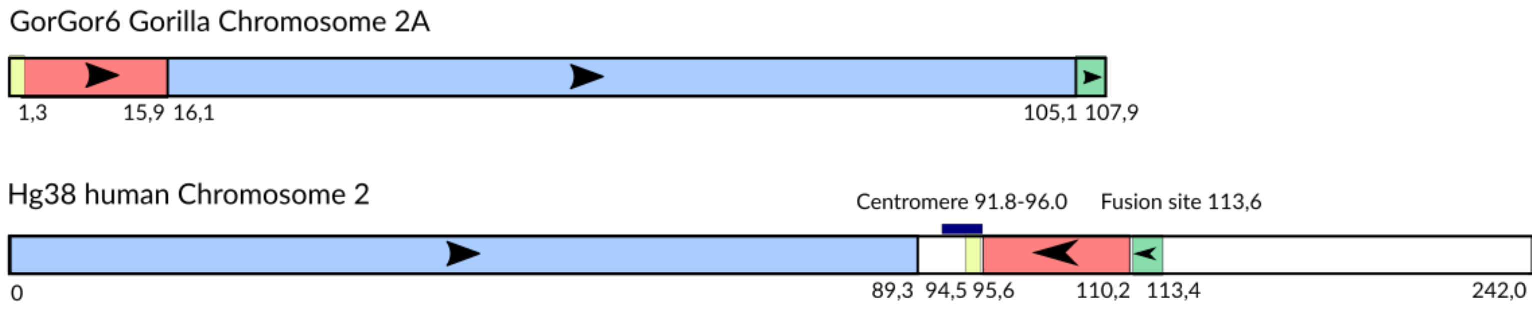

Figure S6: **Example of the GorGor6 reference genome assembly error.** Colours correspond to the syntenic sequences from ChainNet along with their directions and coordinates. Red sequence (approx. 14.6Mb) is a centromere flanking region on the HSA2 (hg38) and is separated from the HSA2 fusion site with a green sequence (approx. 3.2 Mb). Note that since the red sequence has undergone an evolutionarily pericentromeric inversion, the blue sequence (approx. 89 Mb) on the GorGor6 reference genome has wrong orientation. This was additionally confirmed by the Bionano Genomics mapping (Supp. Fig. 4A)

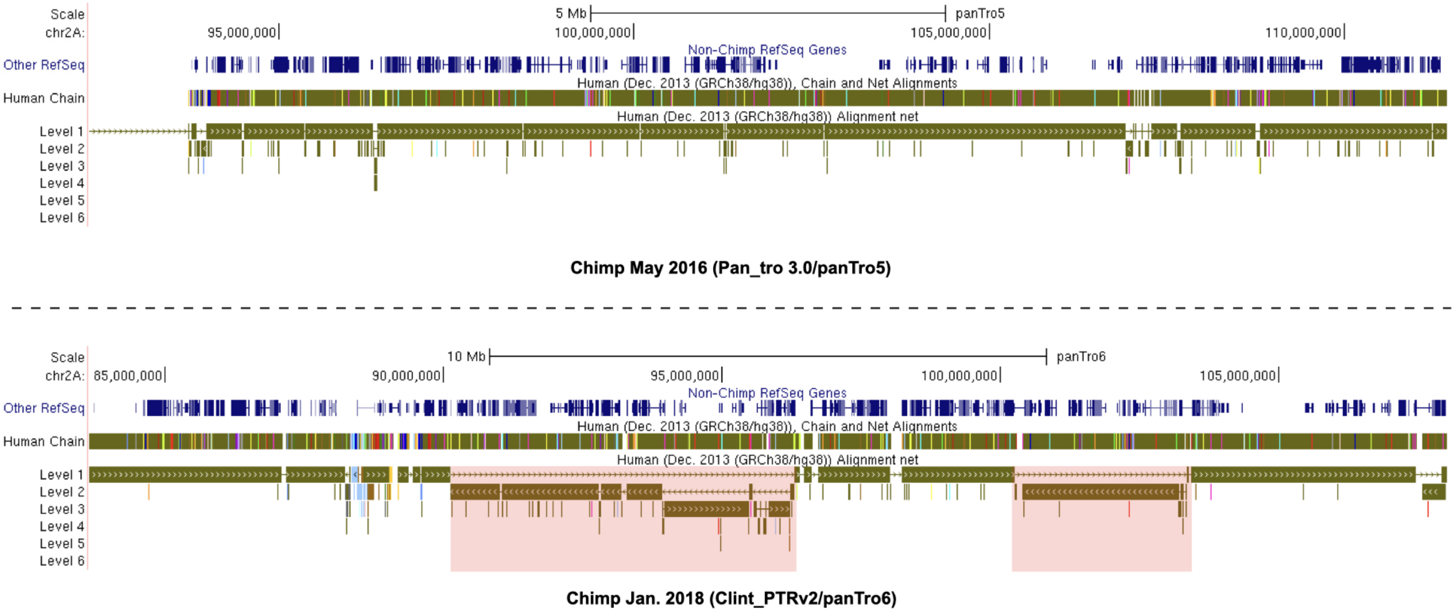

Figure S7: **Comparison of the q arm of chromosome 2B in PanTro5 and PanTro6.** The results of our analysis suggest that the rearrangements marked by the pink stripes are false positives.

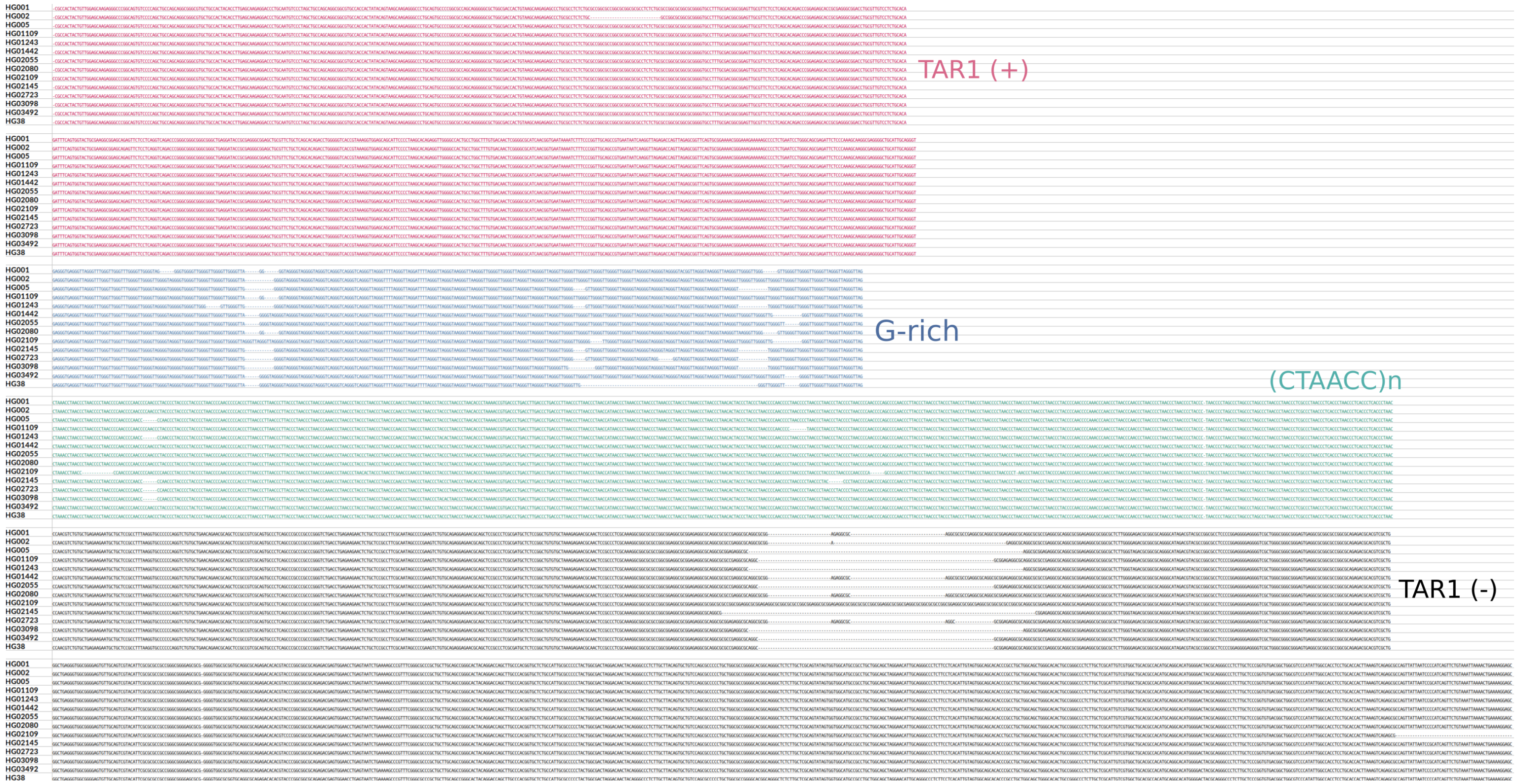

Figure S8: **Multialignment of the genomic fragments flanking the HSA2 fusion site**. Fragments of the assembled contigs flanking the fusion site for 13 human genomes were multialigned by CLUSTALW algorithm with default parameters. For the alignment fragments of the assembled fusion sites contigs were annotated by RepeatMasker (Supp. Tab 1). Fragments subsequently annotated as TAR1 satellite, G-rich low-complexity region, (CTAACC)n simple repeat, and inverted TAR1 satellite was used for the multialignment. In the multialignment visualisation, each region is depicted with a distinct colour.

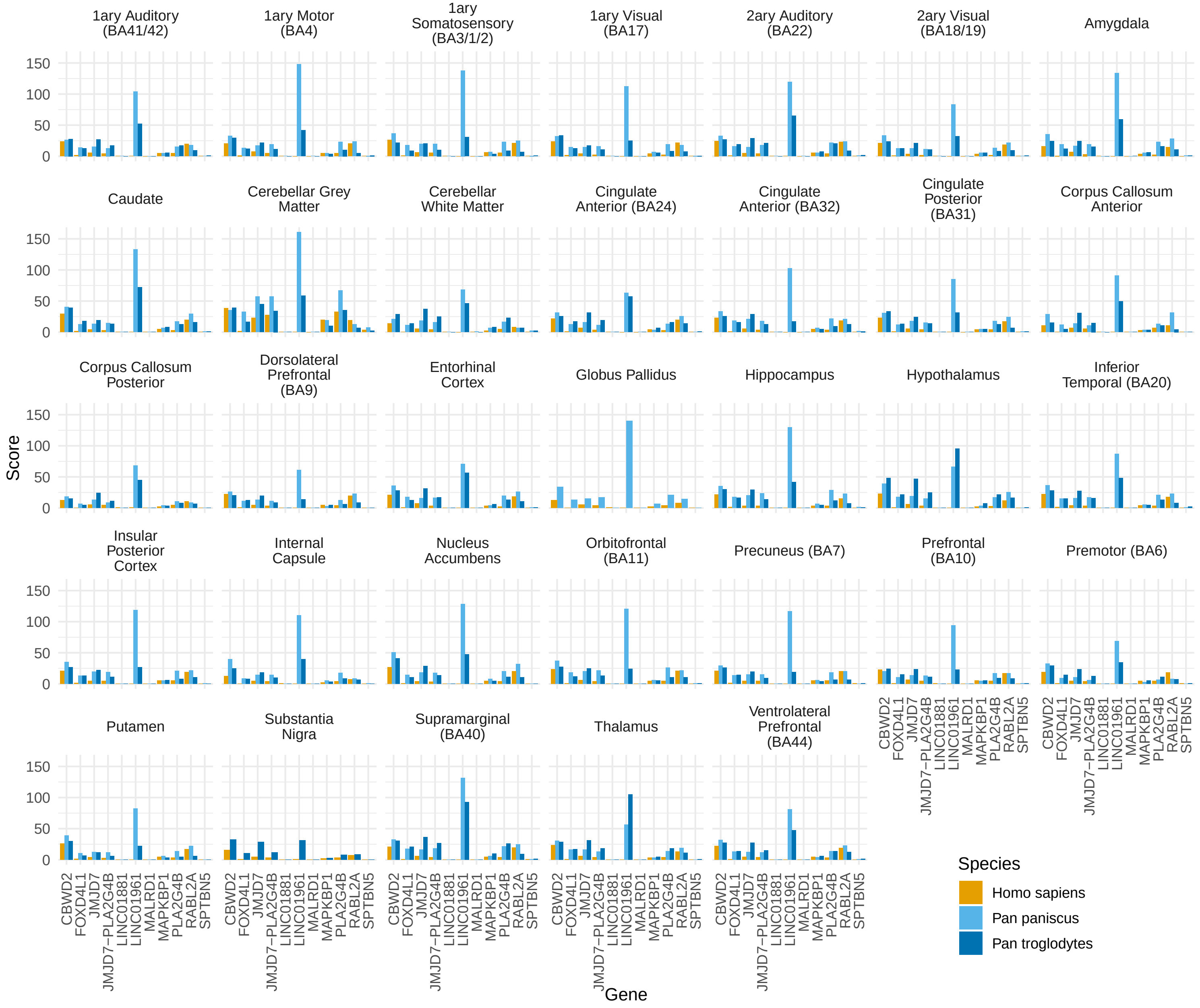

Figure S9: **Expression levels of 11 transcripts in chimpanzee, bonobo, and human (*CBWD2*, *FOXD4L1*, *JMJD7*, *JMJD7-PLA2G4B*, *LINC01881*, *LINC01961*, *MALRD1*, *MAPKBP1*, *PLA2G4B*, *RABL2A*, and *SPTBN5*) found on the extensions of the subtelomeric regions assembled with PhaseDancer.** No data available for: Substantia Nigra (Pan paniscus), Globus Pallidus (Pan troglodytes).

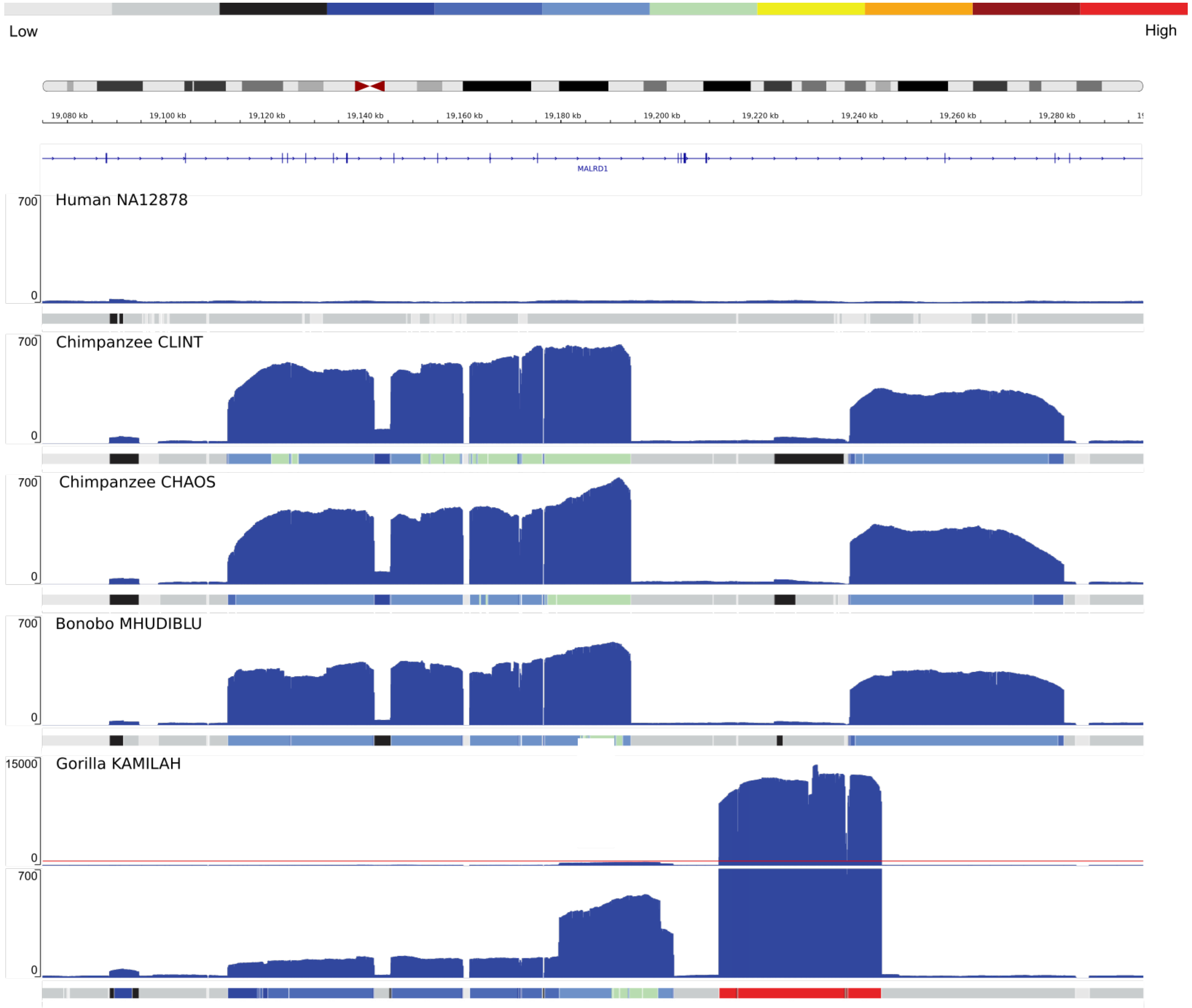

Figure S10: **Normalised depth-of-coverage histogram of the aligned whole-genome CCS reads of a 225-kbp region of human chromosome 10 (chr10:19075000-19300000, NCBI hg38) in human (NA12878), two chimpanzees (Clint, Chaos), bonobo (Mhudilbu) and gorilla (Kamilah).** This region is segmentally duplicated in the chimpanzee, bonobo and gorilla mainly in subtelomeres. In gorilla, two depth-of-coverage tracks are shown. The Y-axis limit of the top track allows for the presentation of all data. The Y-axis limit of the bottom track allows for the presentation of values apart from the region with extremely high coverage. Red line on the top track marks the Y-axis limit of the bottom track.
