# Supplementary material for: PhaseDancer: a novel targeted assembler of segmental duplications unravels the complexity of the human chromosome 2 fusion going from 48 to 46 chromosomes in hominin evolution
Source: Genome Biol. 2023 Sep 11;24:205. doi: 10.1186/s13059-023-03022-8 (PMC10496407; doi:10.1186/s13059-023-03022-8)
Supplement: Supplementary file 2 — Additional file 2. Review history. [file 13059_2023_3022_MOESM2_ESM.docx]

Review History

**First round of review**

**Reviewer 1**

**Were you able to assess all statistics in the manuscript, including the appropriateness of statistical tests used?**

There are no statistics in the manuscript.

**Comments to author:**

The authors present a method, PhaseDancer, to assemble missing duplicated sequences in long-read assemblies. The algorithm was shown to work on simulated data, then applied to resolve a complex region in primate genomes. The method is designed to work on data with high error rates, but as of this submission there are no primate genomes with high coverage high-accuracy data. Extensive testing was performed on BAC and simulated data, and the primate genomes are compared to optical mapping. The ideas here clearly improve the analysis of legacy pacbio data, and are likely to be useful for new data.

Comments:

If the method were applied to Oxford Nanopore data, this could increase the use of the method. There are many genomes being sequenced with this, and the accuracy is lower than PacBio HiFi so the PhaseDancer method could help assemblies with this data.

The algorithm is presented at a high level and is difficult to understand exactly what is done. Can more details be given in the Random Forest Classifier?

Primate analysis:

"Using PhaseDancer in combination with OGM, we confirmed that the regions syntenic

to the HSA2 fusion site correlate well with the latest orangutan genome build

(Supp. Fig. 2)."

This is a vague statement. A more quantitative metric for agreement should be stated.

The simulations that were performed were on haploid genomes, however the primate genomes are of course diploid. Evidence should be provided that the genomes that are recovered do not have structural differences between haplotypes.

Minor question: are the same parameters used for all the primate genomes?

The software seems a bit cumbersome - the readme required many steps to set up. Ideally one could have a bam and an interval in the bam (or multiple intervals), and you just run PhaseDancer on them.

**Reviewer 2**

**Were you able to assess all statistics in the manuscript, including the appropriateness of statistical tests used?**

There are no statistics in the manuscript.

**Comments to author:**

The manuscript "From 48 to 46 chromosomes: a novel targeted assembler of segmental duplications unravels the complexity of the HSA2 fusion" presents a method for targeted assembly of complex regions of the genome, such as segmental duplications, by using a previously assembled contig and iteratively extending the region by re-assembling long reads that map to the contig. The authors validated the method on human BAC clones, and also showed in silico verification and benchmarking (Table 1). The authors then used the method to analyze the human chromosome 2 fusion site, together with the orthologous regions in great apes. The authors provided the tool for scientists to download and the method is of interest to the field due to the current challenges in resolving complex genomic regions such as segmental duplications.

I have the following major comments:

1) There are missing details on how PhaseDancer was validated on human BAC clone sequences. What were the initial contigs that were used in this analysis? Out of 341 BAC clones, how many were considered to be rich in SD regions?

2) I am not convinced that a fair comparison was done with other assemblers in the section "In silico verification and benchmarking" (with results presented on Table 1). The runtime for the other assemblers involve de-novo assembly, while runtime for PhaseDancer does not involve assembly of its starting contig. PhaseDancer leverages previously assembled contigs so this should be considered when comparing runtimes.

3) When running the other tools, did the authors try to tweak the parameters to help the other tools perform better? What were the parameters used to run the other tools? Suppose 90X coverage was used to create the PhaseDancer simulated SD regions and reads, will the other tools perform better? These comments (this comment, #3 and #2 above) are not a critique on the performance of PhaseDancer, which seems to do a good job in all 10 topologies.

4) The authors presented lots of analyses showing that there are many segmental duplications in the human genome (Fig. 2), but did not spend the same amount of effort to show how much of these SDs can be resolved by PhaseDancer. There seems to be some hints on this in Fig. 4B, but it's not clear what analysis was done on Fig. 4B, and in fact only the run time was emphasized on Fig. 4B.

5) In the main text, Figure 4 was mentioned in passing as: "Additionally, we broadly assessed the PhaseDancer performance on in silico reads of various properties provided by PhaseDancerSimulator (Figure 4)." There are many information in Fig. 4 that can't be understood from the caption and should be described better (either in the caption or main text).

6) In Fig. 1. How can PhaseDancer be used for fast personalised diagnostics of SDs? Will the whole genome of the patient be sequenced, or will it involve a targeted sequencing of the region of interest? How deep is the sequencing coverage needed to do this analysis?

7) Analysis of the HSA2 Fusion Event: the authors provided a nice figure showing the syntenic regions in great apes and human, but without seeing the results on how PhaseDancer analyzed these regions (the results could be in the supplementary figures/tables, which are not available in the reviewer website), it is hard to understand what is going on. There seem to be some PhaseDancer results included in Figure 3, i.e., the green stripes, but it's not clear why there are no green stripes for all of the species/contigs. Did PhaseDancer fail on the contigs without green stripes? Or is it because PhaseDancer result showed that there's nothing to extend?

Minor Comments

1) Second sentence of the abstract should be written better — where is the region anchored to? what is being anchored?

2) The captions of the figures are difficult to understand. For example, caption of Fig. 4C is difficult to understand.

3) In the main text, supplementary figures and supplementary tables were mentioned, but I could not download them from the Genome Biology Manuscript website. Thus, I was not able to view any of the supplementary information mentioned in the text.

4) The Methods section on "Clustering phase" is difficult to understand. What are cis-morphisms? Were they ascertained from the reads or from the contigs, or from an external set of SNPs? In the first paragraph of this section, it is hard to understand what sequences are being discussed (the contig? the reads?)

5) About in silico benchmarking, what was the reason for adding random sequences at the beginning and ends of the simulated SDs? What is meant by the "beginnings of each collapsed SD used as the initial sequence for the assembly process "(how long was the initial contig used?, what is the motivation behind this)?

6) The genomes of primates are not as well assembled and annotated (especially for for segmental duplications) as the human genome. This tool has the potential to help improve the assembly and SD annotation of primate genomes, and perhaps the authors could discuss how the tool can be used for this purpose.

**Authors Response**

**Point-by-point responses to the reviewers’ comments:**

Reviewer #1:

The authors present a method, PhaseDancer, to assemble missing duplicated sequences in long-read assemblies. The algorithm was shown to work on simulated data, then applied to resolve a complex region in primate genomes. The method is designed to work on data with high error rates, but as of this submission there are no primate genomes with high coverage high-accuracy data. Extensive testing was performed on BAC and simulated data, and the primate genomes are compared to optical mapping. The ideas here clearly improve the analysis of legacy pacbio data, and are likely to be useful for new data.

Comments:

If the method were applied to Oxford Nanopore data, this could increase the use of the method. There are many genomes being sequenced with this, and the accuracy is lower than PacBio HiFi so the PhaseDancer method could help assemblies with this data.

*Response: We want to thank the reviewer for this comment and in response, we would like to address two significant aspects. Firstly, we demonstrate the effectiveness of PhaseDancer in resolving the OPN1LW/OPN1MW gene cluster in one individual from the T2T diversity panel using Ultra Long Oxford Nanopore data. This serves as an illustration for potential clinical application of PhaseDancer to Oxford* *Nanopore sequencing data. In addition, we would also like to emphasise that, for the purpose of this manuscript, PhaseDancer was employed on PacBio CLR data (as evidenced by the validation on BAC data). Please note that PacBio CLR data are characterised by lower accuracy compared to PacBio HiFi data. Furthermore, we want to underline that the PhaseDancer Simulator has the capability to generate ONT-like data with specific parameters (detailed at https://github.com/bposzewiecka/phaseDancerSimulator; parameter: chemistry {R103, R95, R94} for Nanopore sequences). This feature enables simulation of data resembling that obtained from Oxford Nanopore Technology.*

The algorithm is presented at a high level and is difficult to understand exactly what is done. Can more details be given in the Random Forest Classifier?

*Response: To better clarify the idea behind the clustering procedure we reformulated the corresponding section as follows:*

*The HPC (homopolymer-compressed) reads overlapping the full HPC anchor sequence are selected using the .bam alignment file from the previous step. Using this alignment mismatches are analysed to find candidates for cis-morphisms. Here, a cis-morphism refers to a single nucleotide difference between two or more segmental duplications.*

*To detect cis-morphisms, the frequency of the second most common nucleotide is computed for each locus. A locus is identified as a cis-morphism when the corresponding second most common nucleotide frequency is greater than a given threshold value (parameter dependent on sequencing technology and the coverage). Additionally, when the number of the identified cis-morphisms is greater than a given upper-bound (by default set to 200), only those with the largest percent of the second most common base are retained. Such filtered cis-morphisms are then used for clustering.*

*The first step of clustering is based on the graph connectivity analysis. A graph used for clustering reads is called a similarity graph. A set of vertices of the similarity graph corresponds to the reads overlapping the full HPC anchor sequence. Each edge of the similarity graph connects vertices most similar to each other according to a Hamming distance of cis-morphisms (≤ 0. 4). The decomposition of the similarity graph into the connected components corresponds to the partition the computed reads.*

*In the second step, each block in the partition is subdivided into clusters using cis-morphisms derived from the reads composing the block. The clustering process is based on random simulations and generates multiple alternative clusterings of reads.*

*In each simulation, a random cis-morphism is selected iteratively to partition the set of all reads based on the observed nucleotides. The procedure is applied recursively until either no cis-morphisms are present in the processed set of reads, or the number of reads in each constructed cluster falls below a certain threshold (this threshold is sequencing technology-dependent, yet it is assumed to be 0.8 ⨉ coverage).*

*Given all the alternative clusterings of reads, we assign the best clustering to each block. To evaluate the quality of a clustering, we compute the sum of distances between each read and its nearest cluster. In this context, the distance between a read and a cluster is determined by the Hamming distance between the read and the consensus sequence derived from all reads in the cluster. The best clustering is the one that minimises the sum of distances across all reads.*

*The final clustering of all reads is a union of all clusters from all blocks. The cluster used for the extension of the anchor sequence maximises the number of reads shared with the cluster selected for the extension in the previous iteration. In particular, in the first iteration, the cluster is selected by the similarity to the initial sequence (i.e. Hamming distance between the sequence and clusters consensuses).*

Primate analysis:

"Using PhaseDancer in combination with OGM, we confirmed that the regions syntenic

to the HSA2 fusion site correlate well with the latest orangutan genome build

(Supp. Fig. 2)."

This is a vague statement. A more quantitative metric for agreement should be stated.

*Response: The figures presented in the manuscript were generated using BioNano AccessⓇ software, which enabled visual inspection of the assembled regions. The .xmap files, visualised using this software, did not reveal any structural variations in molecules aligned to the assembly in the region syntenic to the HSA2 fusion site.*

*To clarify which technique confirms the success of the assembly we reformulated the phrase:*

*Using PhaseDancer in combination with OGM, we confirmed that the regions syntenic to the HSA2 fusion site correlate well with the latest orangutan genome build (Supp. Fig. 2).*

*to:*

*"Using PhaseDancer in combination with OGM, we confirmed that the regions syntenic to the HSA2 fusion site map to the latest orangutan genome build showing any structural variations (Supp. Fig. 2)."*

*To not suggest that any quantitative measure was applied to draw these conclusions.*

The simulations that were performed were on haploid genomes, however the primate genomes are of course diploid. Evidence should be provided that the genomes that are recovered do not have structural differences between haplotypes.

*Response: During our analyses and validations, we took into consideration the diploid nature of the primate genomes under investigation. We have addressed this issue in the supplementary materials accompanying our manuscript (see Supp. Fig. 4D) where we visually confirm that there are no structural differences between haplotypes on Chr2A (PanTro5) and Chr2B (PanTro6) using BioNano Access^Ⓡ^ technology.*

*Nonetheless, to address this comment in a broader context we also performed extended validation to confirm the absence of structural differences on other chromosomes. The results of this validation are presented in the figures below, which show the analysis performed on the chimpanzee and bonobo genomes. The only* *chromosome for which a structural variation can be observed is Chr12 (PanTro6), which is due to the expansion of tandemly repeated StSats sequences on one of the haplotypes. These validation results are also added to the supplementary materials.*

*All the figures above are expected to be read in pairs (two figures per chromosome: existing assembly and new assembly provided by PhaseDancer). For every single subfigure there are three stripes: two blue stripes correspond to haplotypes, while a green stripe is related to the assembly. Additionally, vertical lines indicate the cleavage sites of restriction enzymes (expected to be aligned across all three stripes to confirm the correctness of an assembly). Above all green stripes one can read the total length of the* *presented assembly. Note that in each case PhaseDancer provides an extension of the current assembly. As the only exception can be considered Chr9, PanTro6, however, in this case thanks to PhaseDancer we have filled in the 40kb gap that was not assembled in the current reference genome.*

Minor question: are the same parameters used for all the primate genomes?

*Response: Essentially, yes, the parameters depend on the sequencing technology and the coverage, but not on the species analysed. The only information that can be considered as a parameter that came from primate genomes is the initial sequence extended during the assembly process.*

The software seems a bit cumbersome - the readme required many steps to set up. Ideally one could have a bam and an interval in the bam (or multiple intervals), and you just run PhaseDancer on them.

*Response: Here, we would like to extend our appreciation to the reviewer for providing this valuable comment, as it will undoubtedly enhance the user experience. To address this concern, we have incorporated into PhaseDancer an additional, more user-friendly interface. As a result, it is now possible to execute the software directly from the command line using a minimal set of required parameters, which are outlined below.*

*Command line usage of PhaseDancer now looks as follows:*

*Now, five parameters need to be specified to run PhaseDancer:*

*- sequencing technology used to generate the data (one of the following: --pb-clr, --pb-hifi, --ont; unnamed, first positional parameter)*

*- file with all sequencing data from the indicated technology in one of the available formats (.bam, .fastq, fasta; unnamed, second positional parameter)*

*- file with initial anchor sequences in one of the available formats (.fasta, .bam; --initial-anchor-sequences)*

*- coverage of the provided sequencing data (--coverage)*

*- path to the directory where all the output data is expected to be stored (--output-dir)*

*All other parameters are optional with their default values. For the convenience of the reader we attach the printout of PhaseDancer’s help page.*

*Local segmental duplications assembly using PhaseDancer.*

*PhaseDancer is a tool that extends the initial sequences provided by the user to generate larger contigs using a greedy algorithm. Input reads can be provided in FASTA, FASTQ, or BED format, with FASTA and FASTQ files also being able to be compressed with gzip. Currently, PhaseDancer supports PacBio (CLR, HiFi) and ONT reads.*

*The initial sequences can be specified using a BED or FASTA file that describes or contains sequences of equal size. If a BED file is used, the corresponding reference sequence should also be provided. Users should specify the estimated coverage and the number of iterations for the algorithm. The algorithm can extend the provided initial sequences using multiple CPUs. The default contig extension size can be adjusted.*

*Additionally, users can override the parameters of the assembly for each contig using a ".yaml" file.*

**Reviewer #2:**

**Comments to author:**

The manuscript "From 48 to 46 chromosomes: a novel targeted assembler of segmental duplications unravels the complexity of the HSA2 fusion" presents a method for targeted assembly of complex regions of the genome, such as segmental duplications, by using a previously assembled contig and iteratively extending the region by re-assembling long reads that map to the contig. The authors validated the method on human BAC clones, and also showed in silico verification and benchmarking (Table 1). The authors then used the method to analyze the human chromosome 2 fusion site, together with the orthologous regions in great apes. The authors provided the tool for scientists to download and the method is of interest to the field due to the current challenges in resolving complex genomic regions such as segmental duplications.

I have the following major comments:

1) There are missing details on how PhaseDancer was validated on human BAC clone sequences. What were the initial contigs that were used in this analysis? Out of 341 BAC clones, how many were considered to be rich in SD regions?

*Response: Since the flow of PhaseDancer requires initialization with a short sequence, we utilised the flanking sequence of each BAC.*

*To assess the enrichment of BAC clones in the SD region we have mapped BAC clone sequences to the T2T genome assembly. Using the data from the “SEDEF Segmental Dups” track on UCSC genome browser we have computed the average number of stacked SDs for each BAC clone analysed. We have considered BAC clones as enriched in SDs when the number of SDs is above 0.5. Therefore, 283 of 341 (83 %) BAC clones can be considered as rich in SD regions.*

*For different assemblers each histogram below displays the number of BAC tasks (y-axis) given the number of stacked segmental duplications within a given BAC sample data (x-axis). The colour indicates the resolved (blue) or non-resolved (yellow) assembly tasks. Please see also Fig. 2c to compare the abundance of the stacked segmental duplications in the human genome and Fig. 4b to assess the PhaseDancer’s performance for the human genome related assembly tasks.*

2) I am not convinced that a fair comparison was done with other assemblers in the section "In silico verification and benchmarking" (with results presented on Table 1). The runtime for the other assemblers involve de-novo assembly, while runtime for PhaseDancer does not involve assembly of its starting contig. PhaseDancer leverages previously assembled contigs so this should be considered when comparing runtimes.

*Response: In relation to the runtime of the assembly task, we denote the exceeded computation runtime when the process did not finish after 5 days. Analogous scenario was observed across all assemblers utilised for resolving SDs from the data involving more complex topologies generated by PhaseDancerSimulator. Moreover, it is worth noting that all of the simulated assembly tasks are based on rather tractable data. The root sequence used for the generation of the collapsed segmental duplication was 550 Kb long, so the total amount of all simulated collapsed duplications to be assembled did not exceed 8 Mb across all considered topologies.*

*The reviewer is correct in noting that PhaseDancer is provided with an initial contig, which can be viewed as a step to facilitate the assembly process. However, we believe that the aforementioned constraints related to all benchmarking tasks should not be regarded as unattainable for the tested assemblers, particularly those specifically designed for segmental duplication analysis.*

*Apart from the mentioned initial contig, all the data used in our study are simulated and each algorithm is provided with the exactly the same input, thereby eliminating any favourable setting towards PhaseDancer in terms of runtime. Specifically, each algorithm initiates a de novo assembly process on the exactly same set of data.*

3) When running the other tools, did the authors try to tweak the parameters to help the other tools perform better? What were the parameters used to run the other tools? Suppose 90X coverage was used to create the PhaseDancer simulated SD regions and reads, will the other tools perform better? These comments (this comment, #3 and #2 above) are not a critique on the performance of PhaseDancer, which seems to do a good job in all 10 topologies.

*Response: To address this problem, we have investigated two main parameters: coverage and the percent of cis-morphisms. First, we have observed that for a predetermined low level of cis-morphisms, elevating coverage up to 90x did not enhance the efficacy of the tested assemblers. Subsequent investigations aimed at improving the performance of the other assemblers have led us to the conclusion that the number of cis-morphisms was the critical parameter.*

*To assess the impact of the number of cis-morphisms we have simulated the simplest possible topology of the collapsed SDs, i.e. involving exactly two collapsed SDs. Each collapsed SD portion spanned a length of 550 kb and was flanked by the unique random sequences of 50 kb in size, both at the beginning and the end. We have simulated such SD with the parameters defining the expected number of cis-morphisms per 100 bp ranging from 1 to 9 and simulated reads covering such sequences. These reads were used as an input for the Flye and Wtdbg2 assemblers. For each contig generated by the Flye and Wtdbg2 assemblers, we have found the best scoring alignment to two of such sequences. We have visualised these alignments for each assembler and each considered expected number of cis-morphisms per 100 bp. The plot depicts two generated sequences, where the random sequences are in violet, cyan, green and orange and the collapsed SD portions are in blue. Each red line corresponds to the best alignment of each assembled contig on the one of the simulated sequences.*

*From the figure below one can assess the impact of increasing the expected number of cis-morphisms per 100 bp, ranging from 1 to 9, on the assembly of two datasets is presented below. It can be observed that both Flye and Wtdgb2 require around 6-7 cis-morphisms per 100 bp to generate the expected outcome,* *while PhaseDancer solves the problem even for the number of cis-morphisms as low as 1 per 100 bp (see Fig. 5 in the main article).*

*It is worth emphasising that one of the figures presented in the manuscript (shown below) describes the abundance of SDs with specific properties (total size in bp, OY; percent of identity, OX)*

4) The authors presented lots of analyses showing that there are many segmental duplications in the human genome (Fig. 2), but did not spend the same amount of effort to show how much of these SDs can be resolved by PhaseDancer. There seems to be some hints on this in Fig. 4B, but it's not clear what analysis was done on Fig. 4B, and in fact only the run time was emphasized on Fig. 4B.

*Response: We thank the reviewer for this comment. Indeed, Figure 2. was expected to provide an insight for the reader on how abundant is the problem of SDs in the scale of the human genome. Our approach to cover this abundance was to incorporate the BAC clone tasks in the validation procedures. Since the BAC clone validation data are composed of human genome regions enriched in segmental duplications, the performance of PhaseDancer on the human genome is well-assessed. Additionally, the comparison of PhaseDancer to the other assemblers was already covered in the first question.*

5) In the main text, Figure 4 was mentioned in passing as: "Additionally, we broadly assessed the PhaseDancer performance on in silico reads of various properties provided by PhaseDancerSimulator (Figure 4)." There are many information in Fig. 4 that can't be understood from the caption and should be described better (either in the caption or main text).

*Response: We apologise citing the wrong reference to the figure in the pointed sentence (was Figure 4, should be Fig. 4C). In subpanel 4C, as stated in the caption, we have evaluated PhaseDancer performance for different sequencing error levels (x-axis) and different levels of cis-morphisms per 10 kb window (colours). To clarify more, we have now reformulated caption 4C as follows:*

*The evaluation of PhaseDancer assemblies using the Phred Quality Score (Q; y-axis). The samples used for evaluation were generated by PhaseDancerSimulator, with fixed parameters including a coverage of 40x, an average read length of 18 kb, and a read length standard deviation of 3 kb. The x-axis represents different sequencing error levels, while the colour scale indicates different numbers of cis-morphisms per 10 kb window. The additional upper panel in the figure shows the percentage of assembly tasks with no errors (Q > 60) using bar plots. Remarkably, our analysis revealed no significant changes in assembly quality for different PhaseDancerSimulator topologies (SDs evolutionary scenarios).*

6) In Fig. 1. How can PhaseDancer be used for fast personalised diagnostics of SDs? Will the whole genome of the patient be sequenced, or will it involve a targeted sequencing of the region of interest? How deep is the sequencing coverage needed to do this analysis?

*Response: One potential illustration of personalised diagnostics pertaining to the assembly of SD-rich regions can be derived from the research conducted by Haer-Wigman et al2 . Specifically, the authors report on the OPN1LW/OPN1MW gene cluster that is composed of the SDs, one the OPN1LW gene, and one to five OPN1MW genes. We note that the analysis of the OPN1LW/OPN1MW gene cluster necessitates a reference-free de novo assembly, thereby complicating the analysis. Through our examination of a human genome of an individual HG02955 sequenced by Oxford Nanopore Technology, we have demonstrated that PhaseDancer facilitates targeted assembly of the OPN1LW/OPN1MW gene cluster.*

*Using PhaseDancer we have assembled a region with tandem triplication, while in the reference genome hg38 this region consists of a tandem quadruplication (see two dot plots below). Here, we want to emphasise that PhaseDancer managed to distinguish clusters based only on a few cis-morphism per 10 kb anchor sequence.*

*Moreover, we have used the reads that maps on the OPN1LW/OPN1MW gene cluster to assemble this region by wtdbg2 assembler and Flye. Wtdbg2 assembler collapsed segmental triplication into one, while Flye produced contig with triplication, but not flanked with a unique sequence from one side.*

*Moreover, we consider this example to be a showcase of PhaseDancerViewer utility in the examination of clusters of reads that map to a certain region. PhaseDancerViewer application visualizing assembly iterations of the region of OPN1LW/OPN1MW gene cluster for HG02055 genome can be found at:* [*http://bioputer.mimuw.edu.pl:82*](http://bioputer.mimuw.edu.pl:82)*.*

*PhaseDancer can also be used for targeted sequencing of the region of interest or a panel of regions of interest. Currently, we sequence subtelomeric regions by Cas9 enrichment Oxford Nanopore Technology to find structural variants and estimate the length of telomere tracts. Results of these experiments will be analysed using PhaseDancer (data not published).*

*Here, we would like to clarify that PhaseDancer is indeed a specialised tool designed for targeted genome assembly tasks. Its unique focus on regions enriched with segmental duplications makes it particularly valuable for understanding complex genomic structures. That is why, we acknowledge that PhaseDancer holds potential for translational and personalised medical applications and diagnostics; however, at its current stage, additional refinement can enhance the robustness of PhaseDancer, enabling it to meet the rigorous standards and requirements for clinical applications.*

*We are fully aware of the further improvements that can be incorporated in PhaseDancer. One important direction for enhancement is the introduction of assembly polishing using either the long reads remapping onto the assembly or incorporating additional data such as short Illumina reads. By doing so, we aim to enhance the final results provided by PhaseDancer and enable its more robust integration into clinical workflows. Therefore, for now we consider PhaseDancer as a supportive tool in the overview of patient data and hypothesis generation, but before achieving full incorporation in clinical settings further refinement should be done.*

7) Analysis of the HSA2 Fusion Event: the authors provided a nice figure showing the syntenic regions in great apes and human, but without seeing the results on how PhaseDancer analyzed these regions (the results could be in the supplementary figures/tables, which are not available in the reviewer website), it is hard to understand what is going on. There seem to be some PhaseDancer results included in Figure 3, i.e., the green stripes, but it's not clear why there are no green stripes for all of the species/contigs. Did PhaseDancer fail on the contigs without green stripes? Or is it because PhaseDancer result showed that there's nothing to extend?

*Response: We really want to thank the reviewer for this comment, because we hope that the extensions applied to the figure will further enhance demonstration of the potential of PhaseDancer. In the initial version of the Figure, we have only highlighted the PhaseDancer’s extensions for Chr2A and Chr2B in different species (unless the assembly of PhaseDancer was fully consistent with the latest genome build). In the reviewed version of the Figure for each presented chromosome, we have now highlighted all extensions provided by PhaseDancer’s assemblies.*

Minor Comments

1) Second sentence of the abstract should be written better — where is the region anchored to? what is being anchored?

*Response: The abstract was reformulated to meet the limit of 100 words. As a result, the anchoring procedure in the abstract is not mentioned anymore.*

2) The captions of the figures are difficult to understand. For example, caption of Fig. 4C is difficult to understand.

*Response: We have revised all the figures and adapted changes to make them more clear. In particular the caption of Fig 4C was reformulated from:*

*Feasibility limits for samples generated by PhaseDancerSimulator (coverage 40x, read average length 18kb, and read length standard deviation 3kb) given sequencing error (x-axis) and different levels of cis-morphisms per 10~kb window (colour scale). PhaseDancer assemblies were evaluated using Phred Quality Score (Q, y-axis). No error assemblies (Q>60) are summarised in the upper panel. There are no significant assembly quality changes for different PhaseDancerSimulator topologies (SDs evolutionary scenarios).*

*to:*

*The evaluation of PhaseDancer assemblies using the Phred Quality Score (Q; y-axis). The samples used for evaluation were generated by PhaseDancerSimulator, with fixed parameters including a coverage of 40x, an average read length of 18 kb, and a read length standard deviation of 3 kb. The x-axis represents different sequencing error levels, while the colour scale indicates different numbers of cis-morphisms per 10 kb window. The additional upper panel in the figure shows the percentage of assembly tasks with no errors (Q > 60) using bar plots. Remarkably, our analysis revealed no significant changes in assembly quality for different PhaseDancerSimulator topologies (SDs evolutionary scenarios).*

3) In the main text, supplementary figures and supplementary tables were mentioned, but I could not download them from the Genome Biology Manuscript website. Thus, I was not able to view any of the supplementary information mentioned in the text.

*Response: Hereby we apologize for the inconvenience related to the missing supplementary materials. Along with this letter we re-upload the materials, where the reviewers can find the figures and tables mentioned in the main text. The supplementary materials contains the following list of Tables and Figures: - Table 1: RepeatMasker analysis of HSA2 fusion site flanking regions of 3 human genomes from Genome in the Bottle project repository.*

*- Table 2: RepeatMasker analysis of HSA2 fusion site flanking regions of 10 human genomes from T2T Diversity Panel.*

*- Table 3: FOXD4 gene family in the human hg38 genome build.*

*- Table 4: Segmental duplications harbouring FOXD4 gene paralogs in human in the hg38 human genome build.*

*- Table 5: Listing of FOXD4 gene family orthologs locations gene in Great Apes.*

*- Figure 1: Gross inversion events in the course of primate evolution.*

*- Figure 2: Assessment of the current orangutan reference genome quality (ponAbe3) using Bionano Genomics with niking enzymes BSSSI and BSPQI.*

*- Figure 3: Assessment of the current gorilla reference genome quality (gorGor6) using Bionano Genomics, nicking enzyme DLE1.*

*- Figure 4: Assessment of the current chimpanzee reference genomes quality (panTro5 and panTro6) using Bionano Genomics.*

*- Figure 5: Assessment of the current bonobo reference genomes quality (panPan2 and panPan3) using Bionano Genomics.*

*- Figure 6: Example of the GorGor6 reference genome assembly error.*

*- Figure 7: Comparison of the q arm of chromosome 2B in PanTro5 and PanTro6.*

*- Figure 8: Multialignment of the genomic fragments flanking the HSA2 fusion site.*

*- Figure 9: Expression levels of 11 transcripts in chimpanzee, bonobo, and human (CBWD2, FOXD4L1, JMJD7, JMJD7-PLA2G4B, LINC01881, LINC01961, MALRD1, MAPKBP1, PLA2G4B, RABL2A, and SPTBN5 ) found on the extensions of the subtelomeric regions assembled with PhaseDancer.*

*- Figure 10: Normalised depth-of-coverage histogram of the aligned whole-genome CCS reads of a 225-kbp region of human chromosome 10 (chr10:19075000-19300000, NCBI hg38) in human (NA12878), two chimpanzees (Clint, Chaos), bonobo (Mhudilbu) and gorilla (Kamilah).*

4) The Methods section on "Clustering phase" is difficult to understand. What are cis-morphisms? Were they ascertained from the reads or from the contigs, or from an external set of SNPs? In the first paragraph of this section, it is hard to understand what sequences are being discussed (the contig? the reads?)

*Response: The full Clustering Phase section of our article was revised based on the feedback provided also by the first reviewer, please see the introduced changes.*

*In particular, in the section we explicitly defined cis-morphism as follows:*

*Here, a cis-morphism refers to a single nucleotide difference between two or more segmental duplications.*

*To clarify, our article defines cis-morphism as a type of genetic variation. This variation is an extension of the concept of a single nucleotide polymorphism (SNP) and is identified based on a set of segmental duplications and their global alignment. Specifically, each position with a non-homogeneous nucleotide along all aligned sequences, within the considered region, is considered a cis-morphism. This definition approach is similar to the case of the term Segmental Duplication used in the literature instead of the more appropriate term Low Copy Repeat.*

*In the reformulated section we also underline that the cis-morphisms are found using the mismatches in the alignments of reads on the anchor sequence:*

*The HPC (homopolymer-compressed) reads overlapping the full HPC anchor sequence are selected using the .bam alignment file from the previous step. Using this alignment mismatches are analysed to find candidates for cis-morphisms.*

5) About in silico benchmarking, what was the reason for adding random sequences at the beginning and ends of the simulated SDs? What is meant by the "beginnings of each collapsed SD used as the initial sequence for the assembly process "(how long was the initial contig used?, what is the motivation behind this)?

*Response; We have generated random sequences at the beginning of each simulated SD for the sake of simplicity in generation of the initial anchor sequences for the input of PhaseDancer. Initial contigs were 50kb long. However, PhaseDancer could be easily enhanced with the functionality of extending contigs starting from all clusters formed by reads mapped on the initial anchor sequence To clarify the process, we have reformulated the sentence:*

*The beginnings of each collapsed SD were used as the initial sequences for the assembly process.*

*to:*

*Unique random sequences preceding each collapsed SD portion of the generated sequences were used as an initial anchor sequence for the assembly process.*

*Additionally, to provide a better intuition on what we mean by “beginnings of each collapsed SD” one can appreciate a visualisation of the generated sequences in the response to Question 3 of the reviewer.*

6) The genomes of primates are not as well assembled and annotated (especially for for segmental duplications) as the human genome. This tool has the potential to help improve the assembly and SD annotation of primate genomes, and perhaps the authors could discuss how the tool can be used for this purpose.

*Response: In order to justify that the PhaseDancer is applicable to improve the assembly of primate genomes, we present a case-study of patching an exemplary gap on the reference genome of orangutan (ponAbe3) on chromosome 7. We have managed to assemble this region despite quite low local coverage of PacBio reads (~10x). Below the results of BLAT of the assembled contig on ponAbe3 assembly covering the gap and flanking sequences is shown. The initial anchor sequence was obtained from the reference genome, and assembly was produced by extending it rightward.*

*In general, PhaseDancer can be used to patch gaps in the assembly using initial anchor sequences from the existing part.*

*PhaseDancerViewer application visualising assembly iterations of the gap on chromosome 7 of the ponAbe genome can be found at http://bioputer.mimuw.edu.pl:82.*

**Second round of review**

**Reviewer 1**

The authors have addressed my concerns.

**Reviewer 2**

The authors have satisfactorily answered all my comments and I appreciate the effort that the authors made to revise the paper.
